# Monocyte‐derived extracellular vesicles, stimulated by *Trypanosoma cruzi*, enhance cellular invasion *in vitro* via activated TGF‐β1

**DOI:** 10.1002/jev2.70014

**Published:** 2024-11-29

**Authors:** Ephraim A. Ansa‐Addo, Paras Pathak, Maria V. McCrossan, Izadora Volpato Rossi, Mahamed Abdullahi, Dan Stratton, Sigrun Lange, Marcel I. Ramirez, Jameel M. Inal

**Affiliations:** ^1^ School of Human Sciences, Cell Communication in Disease Pathology London Metropolitan University London UK; ^2^ Pelotonia Institute for Immuno‐Oncology, Department of Internal Medicine The Ohio State University Comprehensive Cancer Center Columbus Ohio USA; ^3^ Medical Research Council Harwell Harwell Science and Innovation Campus, Genotyping Core Oxfordshire UK; ^4^ Immunology Unit London School of Hygiene and Tropical Medicine London UK; ^5^ School of Life and Medical Sciences, Biosciences Research Group University of Hertfordshire Hatfield UK; ^6^ Carlos Chagas Institute Fundacao Oswaldo Cruz, (FIOCRUZ‐PR) Curitiba Brazil; ^7^ Postgraduate Program in Cellular and Molecular Biology Federal University of Paraná Curitiba Brazil; ^8^ National Mycobacterium Reference Service‐South (NMRS‐South) Colindale London UK; ^9^ School of Life, Health & Chemical Sciences The Open University Milton Keynes UK; ^10^ Tissue Architecture and Regeneration Research Group, School of Life Sciences University of Westminster London UK; ^11^ University College London, Institute of Women's Health London UK

**Keywords:** cell uptake, endocytosis, extracellular vesicles, *Trypanosoma cruzi*

## Abstract

During cell invasion, large Extracellular Vesicle (lEV) release from host cells was dose‐dependently triggered by *Trypanosoma cruzi* metacyclic trypomastigotes (Mtr). This lEV release was inhibited when IP_3_‐mediated Ca^2+^ exit from the ER and further Ca^2+^ entry from plasma membrane channels was blocked, but whilst any store‐independent Ca^2+^ entry (SICE) could continue unabated. That lEV release was equally inhibited if all entry from external sources was blocked by chelation of external Ca^2+^ points to the major contributor to Mtr‐triggered host cell lEV release being IP_3_/store‐mediated Ca^2+^ release, SICE playing a minor role. Host cell lEVs were released through Mtr interaction with host cell lipid raft domains, integrins, and mechanosensitive ion channels, whereupon [Ca^2+^]_cyt_ increased (50 to 750 nM) within 15 s. lEV release and cell entry of *T. cruzi*, which increased up to 30 and 60 mpi, respectively, as well as raised actin depolymerization at 60 mpi, were all reduced by TRPC inhibitor, GsMTx‐4. Vesicle release and infection was also reduced with RGD peptide, methyl‐β‐cyclodextrin, knockdown of calpain and with the calpain inhibitor, calpeptin. Restoration of lEV levels, whether with lEVs from infected or uninfected epithelial cells, did not restore invasion, but supplementation with lEVs from infected monocytes, did. We provide evidence of THP‐1 monocyte‐derived lEV interaction with Mtr (lipid mixing by R18‐dequenching; flow cytometry showing transfer to Mtr of R18 from R18‐lEVs and of LAP(TGF‐β1). Active, mature TGF‐β1 (at 175 pg/×10^5^ in THP‐1 lEVs) was detected in concentrated lEV‐/cell‐free supernatant by western blotting, only after THP‐1 lEVs had interacted with Mtr. The TGF‐β1 receptor (TβRI) inhibitor, SB‐431542, reduced the enhanced cellular invasion due to monocyte‐lEVs.

## 1 INTRODUCTION

*Trypanosoma cruzi* is a flagellated, intracellular, protozoan parasite of mammals and the aetiological agent of the debilitating Chagas disease in humans. The life cycle of *T. cruzi* alternates between insect vector and vertebrate host (Pérez‐Molina & Molina, 2018). In the insect, *T. cruzi* multiplies as epimastigotes (non‐infective form) before differentiating to metacyclic trypomastigotes (vertebrate infective stage) that are released on the host skin during transmission by the bite of the reduviid (subfamily triatominae) insect. To establish an infection, *T. cruzi* must enter host cells. Metacyclic trypomastigotes (Mtr) then evade innate immune attack (Cestari et al., 2012; Cestari Idos et al., 2009) and infect host cells in order to progress in the life cycle. Once inside host cells, Mtr differentiate to amastigotes (intracellular replicative forms) which after several rounds of division differentiate to bloodstream trypomastigotes. A model for these, termed Tissue‐culture derived trypomastigotes (TCT), can be derived through in vitro cultivation.

The bloodstream trypomastigotes disrupt infected cells and circulate in the blood, infecting other cells or are taken up by the insect vector, thereby restarting the life cycle. Parasite invasion of various cell types is a complex process with many factors involved, but nonetheless three distinct entry mechanisms have emerged (Ferri & Edreira, 2021). The first two coincide with the formation of a tight membranous vacuole in which the parasite transiently resides (Barrias et al., 2013; Rodríguez‐Bejarano et al., 2021): (1) Ca^2+^‐mediated lysosomal exocytosis to the point of entry (20%–30% (Woolsey et al., 2003)) and (2) endocytosis, including microdomain/lipid raft‐mediated (also known as caveolin‐mediated endocytosis), clathrin‐mediated, and macropinocytosis (70%–80% (Woolsey et al., 2003)). The third entry mechanism depends on autophagy (Romano et al., 2009).

An increase in host cell [Ca^2+^]_cyt_ also results in the release from cells of various Extracellular Vesicles (EVs) (Messenger et al., 2018; Stratton et al., 2015; Théry et al., 2018). EVs can be divided into subpopulations based on size, namely small EVs (sEVs, < 200 nm), and large EVs (lEVs, > 200 nm) (Welsh et al., 2024)). Other terms include exosomes which have an endocytic origin and microvesicles (MVs), a term limited to EVs for which the biogenesis pathway has empirically been shown to involve budding from the plasma membrane (PM); they are also termed shedding MVs, ectosomes, microparticles (Raposo & Stoorvogel, 2013) or plasma membrane‐derived vesicles (Grant et al., 2011). EVs are intact membrane vesicles released constitutively or upon stimulation (de Jong et al., 2012; Stratton et al., 2015) and carry a range of miRNA, lncRNA, cytokines, receptor proteins, and bioactive lipids (De Sousa et al., 2022), that are selectively enriched within the nascent EV. The release (ectocytosis) of shedding MVs budding off the cell surface, recently reviewed (Clancy et al., 2021; De Sousa et al., 2022) occurs upon Ca^2+^‐mediated activation of calpain, depolymerisation of the actin cytoskeleton and loss of PM asymmetry (De Sousa et al., 2022). This results in a blebbing of the MV, changes in lipid content inducing membrane curvature, actin‐myosin contraction at the neck of the nascent MV and its eventual pinching off and release into the extracellular space. The range of both pathogen and host cell‐derived EVs play a plethora of roles in infectious disease (De Sousa et al., 2022; Inal et al., 2013; Schorey & Harding, 2016; Schorey et al., 2015).

EVs represent an important means of intercellular communication between parasites and host cells. EV populations affecting Mtr cell entry may be of parasitic origin, or from any nearby host cell the parasite interacts with, including immune cells (Torrecilhas et al., 2020). EVs have many roles in infection, from increasing cellular invasion (Torrecilhas et al., 2020) and inhibiting immune effector mechanisms, for example inhibiting C3 convertase (Cestari et al., 2012), or even enhancing immune effector mechanisms (Macaluso et al., 2023), to affecting host cell growth (Dekel et al., 2021). THP‐1 monocyte‐derived lEVs, which carry TGF‐β1, are important for invasion and in experiments in mice, inhibiting TGF‐β1‐mediated signalling, reduced fibrosis in cardiac tissue (Ferreira et al., 2019). EVs from both parasites and infected cells tend to promote a proinflammatory response (Cortes‐Serra et al., 2022). Besides *T. cruzi* mediating increases in intracellular calcium (Cestari et al., 2012), parasite EVs themselves can provoke similar increases, also affecting the actin cytoskeleton and mediating cell cycle arrest (Moreira et al., 2019). Such increased [Ca^2+^]_cyt_ stimulates cleavage of actin by calpain, inducing EV release from host cells and although all stages of the parasite induce EV release, those that infect mammalian cells are most potent (Ramirez et al., 2017). As intercellular communicative vectors, EVs provide protection to cytokines they carry (Buzas, 2023) but it is still unclear how intravesicular cytokines bind their respective receptors, which are all type I or type II integral membrane proteins (Wang et al., 2009).

*T. cruzi* Mtr has tropism for a wide range of phagocytic and non‐phagocytic cells, with epithelial cells representing the first barrier parasites face in establishing infection. The human epithelial cell line, HeLa, which like many epithelial cells has non‐professional phagocytic capacity, is a widely used in vitro model in the study of cellular invasion with *T. cruzi* (Barrias et al., 2013; Chiribao et al., 2014; Maeda et al., 2012) and an established recipient cell for EV transfer studies (O'Brien et al., 2022; Verdera et al., 2017). We chose to work with HeLa again as it is easily infected (80% of cells, 24 hpi) by a range of parasite strains (Duran‐Rehbein et al., 2014). In this study we investigated the role of parasite‐derived host lEVs, comparing the effect of host epithelial cell‐ (HeLa) and immune cell‐ (THP‐1 monocyte) derived lEVs, in cell entry. We also expanded on previous work describing host‐derived lEVs harbouring TGF‐β1 (Cestari et al., 2012), aiming to elucidate how this cytokine, on monocyte‐derived lEVs, may be activated to enhance cell entry. We aimed to compare this with epithelial cell‐derived lEVs which present lower levels of TGF‐β1.

Overall, we aimed to compare the effect of lEVs from epithelial and monocytic cells on cellular invasion and elucidate how monocyte‐derived lEVs enhance cell entry, particularly through the role of TGF‐β1. We also aimed to dissect the molecular interactions Mtr forms make with host epithelial cells, for example stimulating mechanosensitive channels (MSCs), that lead to lEV release, and to better understand the role of Ca^2+^ in the Mtr‐stimulated release of host lEVs. We also wanted to deduce whether lEV release which accompanies calpain‐mediated actin rearrangement is itself important in the infection process.

## 2 MATERIALS AND METHODS

### 2.1 Antibodies and reagents

The following mouse monoclonal antibodies were obtained from Abcam: anti‐CD49d (clone P4C2), anti‐TSG101 (clone 4A10), anti‐ANXA1 (clone EPR19342), anti‐CD63 (clone MX‐49.129.5), anti‐α‐tubulin (DM1A)), anti‐β actin (clone mAbcam 8226), anti‐CD44 (clone BLR038F), anti‐CAPNS1 (EPR3324) and anti‐TGF‐β1 (clone BLR195J). Annexin V‐Alexa Fluor 488 was purchased from Invitrogen and Annexin V‐FITC from Thermo Fisher Scientific. Calpeptin was obtained from Merck Biosciences. RGD and RGE peptides, and EGTA, were purchased from Sigma‐Aldrich (St. Louis, MO). Nifedipine, Verapamil, GdCl_3_ and nystatin were purchased from Thermo Fisher Scientific. DNA stain, DAPI, was purchased from Sigma Aldrich as were the viability (DNA) stains, propidium iodide and Hoechst. The peptide GsMTx‐4 was obtained from Peptides Int. Inc, USA. BzATP and calcium ionophore (A23187 or calcimycin) were purchased from Sigma‐Aldrich. The endocytosis inhibitors methyl‐β‐cyclodextrin, filipin, and gadolinium chloride were all obtained from Sigma‐Aldrich. TGF‐β1 was detected on the surface of cells using mouse anti‐human Latency Associated Peptide LAP (TGF‐β 1) PE‐conjugated, clone #27232 (R&D Systems). TGF‐βR1 inhibitor, SB‐431542 was from Sigma‐Aldrich.

### 2.2 Mammalian cell culture

The HeLa cells (human cervical adenocarcinoma), THP‐1 monocytes (human acute monocytic leukaemia) and Vero (African Green monkey kidney cells) used in the study were newly purchased from the European Collection of Authenticated Cell Cultures (ECACC)). The cells, authenticated and deemed mycoplasma‐free by ECACC, were maintained in complete growth medium (CGM) containing RPMI‐1640 supplemented with 10% heat‐inactivated fetal bovine serum (FBS), 100 U/mL penicillin, and 100 mg/mL streptomycin. In addition, cells were occasionally maintained for a week in CGM supplemented with 1% kanamycin at 37°C in a humidified atmosphere of 5% CO_2_. Exponentially growing cells were counted and viability determined using the Guava EasyCyte flow cytometer 8HT (ViaCount assay; Guava Technologies, Hayward, CA). Throughout, after 3 days in culture, cells were split 1:4 and only cultures with at least 95% viability were used.

### 2.3 Bacterial culture

*Salmonella enterica* subsp. *enterica* serovar Typhimurium (wild‐type, strain SL1344), (henceforth termed *S. typhimurium*) was obtained from Culture Collections at the UK Health Security Agency, and its non‐invasive form, strain SL1344 carrying a deletion in the SPI1 pathogenicity island (ΔSPI1) was kindly provided by Prof. Jay Hinton (Institute of Food Research, Norwich Research Park, Norwich). *Salmonella enterica* strains (*S. typhimurium* wild type and strain SL1344), were grown aerobically in Luria Bertani (LB) broth and agar (tryptone, 10 g L^−1^; yeast extract, 5 g L^−1^; NaCl, 10.0 g L^−1^; pH 7.0). *Giardia intestinalis* was grown and maintained as described previously (Evans‐Osses et al., 2010).

### 2.4 T. cruzi culture and purification of metacyclic trypomastigote forms

*T. cruzi* epimastigotes, strain Silvio X10/6, were cultured to exponential growth phase in liver infusion tryptose (LIT) liquid medium with 10% heat‐inactivated FBS at 28°C. Metacyclic trypomastigote forms were obtained and purified with slight modifications, according to the method of Sousa et al. (1983) (de Sousa, 1983). Briefly, epimastigotes in the stationary phase were incubated at 28°C for 2 h in Triatomine Artificial Urine medium (TAU; 190 mM NaCl, 17 mM KCl, 2 mM CaCl_2_, 2 mM MgCl_2_, 8 mM phosphate buffer [pH 6.0]) and subsequent incubation in TAU3AAG medium (TAU supplemented with 10 mM L‐proline, 50 mM L‐glutamate, 2 mM L‐aspartate, 10 mM glucose) for 3 days. In some experiments, epimastigotes in the late stationary phase differentiated into metacyclic trypomastigote forms after incubation at 28°C for 5 days in LIT medium. Metacyclic forms were identified under the light microscope, and by DAPI‐staining to observe the position of the nucleus and kinetoplast as well as characteristic size, shape, and motility. Percentage metacyclogeneis was calculated from counting epimastigote and metacyclic forms on a Neubauer chamber and results were confirmed by counting after exposing the cultures to 10% FBS for 4 h, which should lyse any epimastigote forms, but not resistant metacyclic forms. Once confirmed, metacyclic forms were purified on the day of the experiment. For this, purification by ion exchange chromatography, as used previously (Rossi et al., 2022), was chosen over lysis of remnant epimastigote forms. Parasites were harvested by centrifugation (4000 rpm, 10 min; A‐4‐62 swing‐out rotor, using 5810R centrifuge, Eppendorf) and resuspended in Phosphate‐Saline‐Glucose buffer (PSG) (73 mM NaCl, 1% glucose, 5 mM sodium phosphate, pH 8.0). Metacyclic forms were separated in diethylaminoethyl (DEAE)‐52‐cellulose and aliquots of eluates containing > 95% metacyclic trypomastigotes (Mtr) were resuspended in PSG buffer after centrifugation and counted using a haemocytometer. To confirm that Mtr had been obtained, western analysis to show expression of the Mtr‐specific surface marker, gp90, was carried out (Figure S1A).

As Mtr (in the presence of Ca^2+^ (1 mM)) also release EVs that bud from the surface (Figure S1B, C and inset) or through release of MVBs fusing with the PM (Figure S1D, E and inset), care was taken throughout as prescribed recently (Fernandez‐Becerra et al., 2023) to reduce the presence of parasite EVs, by careful washing of parasites, especially before applying to cells to stimulate EV release. Where tissue culture (cell‐derived) trypomastigotes (TCT) were required, these were obtained by infecting Vero cells with purified MTr and maintaining in Eagle's Minimal Essential Medium with 5% (v/v) FBS. After 5 days of development within the cells, TCT were harvested from the growth medium by centrifugation (3500 × *g*/5 min) and washed three times with PBS (to guard against contamination with host EVs) (Fernandez‐Becerra et al., 2023), the number of metacyclic trypomastigotes being determined using a Neubauer chamber. This harvesting procedure was then repeated every 5 days.

### 2.5 Knockdown of CAPNS1 (Calpain Small Subunit 1) by small interfering RNA (siRNA) transfection

GeneSolution siRNA containing four different sequences targeted to specific sites in *CAPNS1* mRNA (GenBank Accession No. X04106.1) and a negative control siRNA were obtained from Qiagen (Qiagen House, Crawley, UK). The siRNA was reconstituted in sterile RNase‐free water at a final concentration of 10 µM. For invasion experiments, HeLa cells (1 × 10^5^/well in sextuplicate) were transfected 48 h prior to performing experiments at a final concentration of 50 nM using HiPerFect transfection reagent (HPF, Qiagen) according to manufacturer's instructions (Qiagen).

The sequence for the human *CAPNS1* siRNAs were: siRNA#1, 5′ ‐CAC CTG AAT GAG CAT CTC TAT ‐3′; siRNA#3, 5′ ‐AAG GTG GCA GGC CAT ATA CAA ‐3′; siRNA#5, 5′ ‐CAG CGC CAC AGA ACT CAT GAA ‐3′; siRNA#6, 5′ ‐TCC GAC GCT ACT CAG ATG AAA ‐3′. Negative control siRNA, 5′ ‐AAT TCT CCG AAC GTG TCA CGT ‐3′. siRNA#6 decreased CAPNS1 expression the most consistently. Therefore, siRNA#6 was used in subsequent experiments to assess the effects of decreasing CAPNS1 levels on the sensitivity of HeLa cells to *T. cruzi* metacyclic invasion.

### 2.6 Immunoblotting analysis

This was carried out as described earlier (Hui et al., 2005; Inal, 1999). Essentially, HeLa cells, transfected or not with *CAPNS1* siRNA, and the purified lEVs as well as Mtr and epimastigote forms were lysed with lysis buffer (100 mM HEPES/KOH, 2 mM CaCl_2_, 0.5% Triton X‐100) containing protease inhibitor cocktail (Sigma‐Aldrich). The protein content of the lysates was quantified using the BCA assay kit (Pierce Biosciences) and 30 µg was resolved by SDS‐PAGE on a 12% acrylamide gel using the Bio‐Rad Mini‐Protean system. Proteins were transferred to nitrocellulose membrane (Amersham Biosciences, GE Healthcare, Buckinghamshire, UK) using a semi‐dry blotting apparatus (Bio‐Rad) and where necessary stained with the reversible protein stain, Swift Membrane Stain (G‐Biosciences). Membranes were then blocked in 3% w/v non‐fat milk in PBS‐0.1%v/v tween20 (PBST) with shaking for 1 h at room temperature.

All antibodies, diluted at 1:500 with 3% non‐fat milk in PBST, were incubated with the membrane for 1 h at RT. After 6 × 10 min washes in PBST, membranes were then incubated with a 1:10,000 dilution of horseradish peroxidase‐conjugated secondary antibody. After washing (6 × 10 min in PBST), protein bands were visualised with the LumiGOLD ECL Western Blotting Detection kit (SignaGen Laboratories, Rockville, MD 20850), and the chemiluminescent signal detected using the ChemiDoc‐It Imaging System (UVP, LLC, Cambridge, UK).

### 2.7 EV size and concentration determination by Nanosight Tracking Analysis (NTA; nanosight)

NTA was carried out as described previously (Antwi‐Baffour et al., 2020; Kholia et al., 2015; Sisa et al., 2019) using the NanoSight NS300 (Malvern Scientific) equipped with a sCMOS camera and 405 nm diode laser. Data was processed using NTA software (version 3.00) and according to the Minimal Information for Studies of EVs (MISEV2018) (Théry et al., 2018) and MISEV2023 (Welsh et al., 2024). Before taking readings, settings for minimum track length and blur as well as particle size were applied including the ambient temperature. The NTA was calibrated using a standard (100 nm diameter polystyrene beads from Malvern Scientific). Keeping to 20–40 particles in each field of view by appropriate sample dilution, six videos (30 s each at camera level 11) were recorded. Before processing, the background signal was subtracted. Each sample was read in sextuplicate with a minimum of 1000 tracks per measurement. For analysis, the same setting (detection limit 3) was adhered to.

### 2.8 Cell treatments and stimulation from host cells of EV release by metacyclic trypomastigotes/sublytic complement deposition

HeLa cells were plated at 1×10^5^ cells/well in CGM into 12‐well plates containing sterilized 18 mm round coverslips and incubated at 37°C in 5% CO_2_ for 24 h. Cells were washed twice with EV‐ and serum‐free RPMI‐1640 without phenol red (EVSF‐RPMI) (rendered EV‐free (Fernandez‐Becerra et al., 2023) by centrifugation: 100,000 × *g*; 90 min) and after 18 h in EVSF‐RPMI, preincubated at 37°C for 30 min in EVSF‐RPMI/CaCl_2_ (1 mM) with various agents including the lipid raft inhibitors (MβCD, 5 mM, filipin, 5 µg/mL and nystatin, 20 µg/mL), calcium channel blockers (GsMTx‐4, 20 µg/mL; GdCl_3_, 200 µM; Verapamil, 200 µM and Nifedipine, 200 µM) and integrin inhibitors (RGD peptide, 100–500 µg/mL; control RGE peptide, 200 µg/mL; mouse anti‐CD49d, 10 µg/mL) and then removed. In some experiments HeLa cells were pre‐treated separately with the calpain inhibitor, calpeptin (20 µg/mL; 10 min). In invasion assays where lEVs (from HeLa epithelial cells or THP‐1 monocytes) were included, these were added as before (Wyllie & Ramirez, 2017) at 2.5 µg protein (10^7^ lEVs/well), (and where needed, were heat inactivated at 65°C for 30 min). lEVs were also collected from THP‐1 cells treated with Mtr, in which case cells were washed to remove non infecting parasites, to avoid contamination with Mtr‐derived EVs (Fernandez‐Becerra et al., 2023). As a control, the parasites used were sometimes heat inactivated (rendering them immotile) at 50°C for 45 min. Cells were then washed with ice‐cold EVSF‐RPMI before treating with Mtr (5:1 parasites:cell ratio) for 10 min at 37°C. For some experiments, the cell culture supernatants were collected for EV analysis by NTA (at various time points).

The procedure to stimulate HeLa cells with sublytic complement for EV release was carried out as described before (Stratton et al., 2015). Essentially, HeLa cells (2 × 10^5^ cells/mL) were pre‐sensitized with 5% v/v rabbit anti‐HeLa cell membrane antiserum in RPMI‐1640 and 2 mM CaCl_2_ (1 h at 4°C). The cells were then treated with 5% EV‐free normal human serum (NHS, type AB, Sigma). The concentrations of anti‐HeLa serum and complement were empirically determined using a checkerboard titration. The sublytic level of complement used was thus established as a treatment of the cells with 5% NHS for 10 min at 37°C having carried out sensitisation with 5% antibody (1 h at 4°C). In addition, EGTA (5 mM) was used in most lEV induction experiments as negative control.

### 2.9 Flow cytometry analysis

Trypsinised HeLa cells (1 × 10^6^/well in triplicate) resuspended in serum‐ and EV‐free‐RPMI (rendered EV‐free by centrifugation: 100,000 × *g*; 90 min) were left untreated or pre‐treated with calpeptin, GdCl_3_, or GsMTx‐4, respectively and washed twice with PBS as described. Cells were resuspended in Ringer's solution (138 mM NaCl, 2.7 mM KCl, 1.06 mM MgCl_2_, 1.8 mM CaCl_2_, 12.4 mM HEPES and 5.6 mM D‐glucose [pH 7.4]) and stimulated by addition of *T. cruzi* metacyclic trypomastigotes at a 5:1 (parasites‐to‐cells) ratio or sublytic complement (5% NHS), and incubated at 37°C for 30 min. Cells were immediately fixed with 4% paraformaldehyde (PFA) after 30 min and stained in the dark with 10 µg/mL anti‐CD63 Alexa Fluor 488 (R&D Systems), diluted in cold PBS with 3% BSA, at 4°C for 1 h with shaking. In some experiments cells were also labelled with 10 µg/mL Annexin V Alexa Fluor 488. For detection of human TGF‐β1 delivered by monocyte lEVs to the surface of Mtr, anti‐LAP(TGF‐β1) was used to detect surface LAP‐TGFβ1. The flow cytometers used were either a FACScan flow cytometer (BD Biosciences), using CellQuest software for data acquisition and analysis, or a Guava easyCyte 12HT microcapillary flow cytometer at a flow rate of 0.56 mL/s and using ExpressPlus software.

### 2.10 Determination of the Globular actin (G actin): Filamentous actin (F actin) ratio (G:F actin ratio) in HeLa cells by flow cytometry

To measure the actin polymerization status of *T. cruzi* infected cells, which as well as affecting cellular infection may affect EV release, we employed a flow cytometric assay, comparing levels of unpolymerized G‐actin, which was specifically stained by DNase I‐Alexa fluor 488, and polymerised filamentous actin (F‐actin) (Figure 3g), stained by phalloidin‐Alexa Fluor 660; previously densitometry of western blots (Mott et al., 2009) has been used. Fixed (4% PFA) and permeabilised (0.1% Triton X‐100) HeLa cells (10^5^) were stained with DNase I, Alexa Fluor 488 Conjugate (Thermo Fisher Scientific) to detect G‐actin and Phalloidin Alexa Fluor 660 Phalloidin (Thermo Fisher Scientific) to detect F‐actin by flow cytometry (FACScan). The respective median fluorescence intensity (MIF) values were used to calculate the G:F actin ratios for infections in the presence of calpeptin.

### 2.11 In vitro invasion assays

Semiconfluent HeLa cells in EVSF‐RPMI were infected in vitro with *T. cruzi* metacyclic forms using a modification of a method described earlier (Ansa‐Addo et al., 2010). HeLa cells (2 × 10^5^/well in a 24‐well plate) pretreated or not with the agents mentioned earlier, and now seeded on round, 12 mm coverslips, after overnight incubation (37°C/5% CO_2_), in EVSF‐RPMI were infected by the addition of PKH26‐labelled *T. cruzi* metacyclic forms at a 5:1 (parasites‐to‐cell) ratio for different times and incubated at 37°C/5% CO_2_ for 60 min. Cells were then washed three times with PBS, fixed for 10 min at RT with 4% PFA and washed a further three times with PBS. The coverslips were mounted on microscope slides with DAPI‐Vectashield medium (Vector Laboratories, Burlingame, CA), and images were collected using a fluorescence microscope (Bright field, IX81 motorized, inverted fluorescence microscope; Olympus). The number of intracellular parasites was determined by counting at least 500 cells on all coverslips; the number of parasites per cell was also determined.

### 2.12 Plasma membrane repair assay

HeLa cells were treated with *T. cruzi* metacyclic forms at a 5:1 ratio (30 min/37°C) in DMEM in the presence or absence of 2 mM CaCl_2_. Cells were stained with propidium iodide (PI) (0.5 µg/mL or Hoechst 33342 (6 µg/mL). PI‐positive cells were either counted by microscopy or determined by flow cytometry (FACScan) with 10,000 events being recorded for each sample.

### 2.13 Purification and characterisation of lEVs from conditioned and non‐conditioned medium (N‐CM)

After preincubation for 1 h in EVSF‐RPMI, cells were pretreated or not with various lEV inhibitors in EVSF‐RPMI/CaCl_2_ (1 mM) as described above. Cells (HeLa or THP‐1 monocytes) were either left unstimulated (control) or stimulated with sublytic complement (5% NHS), *T. cruzi* Mtr, *S. typhimurium* (5:1, bacteria per cell) or *G. intestinalis* (5:1 parasite to cell ratio). After infection, as described earlier, conditioned medium was collected and lEVs were isolated as described previously (Kholia et al., 2015). Briefly, cells were pelleted at 200 × *g* for 5 min and the resulting supernatant then centrifuged twice at 4000 × *g* for 30 min to remove cell debris. N‐CM was treated in the same way to generate FBS‐lEVs. The supernatant was sonicated for 5 × 1 min in a sonicating water bath (Townson and Mercer Ltd, Croydon) to disperse aggregated sEVs and centrifuged at 15,000 × *g* for 2 h to pellet lEVs. Pelleted lEVs were washed once by resuspending in PBS and centrifuging at 15,000 × *g* for 2 h. The lEV pellet (HeLa‐, THP‐1‐ or FBS‐derived) was resuspended in PBS and quantified by NTA as described above. For some experiments, lEVs or sEVs were stained with anti‐CD44‐PE (phycoerythrin) or Annexin V‐FITC to detect PS expression, by flow cytometry.

### 2.14 Isolation of small EVs and sucrose gradient

Supernatant from cell debris‐free conditioned medium used to isolate lEVs was ultracentrifuged at 160,000 × *g* for 16 h to pellet sEVs. Samples were then subjected to sucrose equilibrium centrifugation. Briefly, sEV pellets were resuspended in 2 mL of PBS and transferred to the bottom of a SW41 centrifugation tube (Beckman Coulter, Fullerton, CA). A 10%–40% (w/v) discontinuous sucrose density gradient was layered on top, and the gradient was centrifuged at 30,000 × *g* for 1 h. Fractions of 1 mL were collected from the bottom of the tube, and the density of each fraction was determined using a refractometer (Schmidt and Haensch, Berlin, Germany).

### 2.15 Transmission electron microscopy

Trypsinised HeLa cells (5 × 10^6^/mL) were resuspended in prewarmed (37°C) EV‐ and serum‐free EVSF‐RPMI supplemented with 0.5 mM CaCl_2_. Cells, either stimulated (with 5% NHS or *T. cruzi* metacyclic forms, 5:1 ratio) or not (control), were fixed with 0.1 M fixative solution (3% glutaraldehyde in 0.1 M sodium cacodylate buffer [pH 7.2]). Samples were post‐fixed by incubation at 0°C for 1 h in 1% osmium tetroxide solution (2% osmium tetroxide (Sigma‐Aldrich) at a 1:1 ratio with 0.2 M sodium cacodylate buffer), followed by ×3 washes in deionised water and then block stained by resuspending in 1% aqueous uranyl acetate (with shaking overnight). After two further washes in deionised water, samples were resuspended in 1% hot agarose, dehydrated in an ascending ethanol series (from 70% to 100% absolute ethanol, v/v, 30 min each time) and washed twice (30 min each) with propylene oxide (Agar Scientific, Essex, UK). Sample was infiltrated with a 1:1 mixture of propylene oxide: Agar resins (mixture of 4.8 g agar resin, 3.6 g MNA (E.M. grade), 1.9 g DDSA (E.M. grade) and 0.2 g BDMA, Agar Scientific) and left rocking overnight at room temperature. Infiltrated samples were embedded in capsules using applicators and polymerized at 60°C for 24 h. To visualise *T. cruzi* Mtr spontaneously releasing EVs, parasites were washed in RPMI‐1640 (2500 × *g*) and incubated (2 h/37°C) in RPMI‐1640 (1 × 10^8^/mL). They were then fixed and stained as described above for monitoring EV release from HeLa cells.

In other experiments, pure isolated lEVs and sEVs were separately fixed and infiltrated with agar resin. Ultrathin sections were cut on a Leica Ultracut R ultra microtome (Leica Microsystems, Deerfield, IL), picked up onto pioloform copper grids and stained for 10 min in Reynolds lead citrate stain. After washing in ultrapure MilliQ water, the sections were examined on a Jeol JEM – 1200 Ex II electron microscope (JOEL, Peabody, MA).

For negative staining, lEVs were stained with 2% aqueous uranyl acetate or 2% phosphotungstic acid (pH 6.8) plus aqueous bacitracin. Samples were placed on 400 mesh copper grids with a Pioloform support film (grids and Pioloform powder from Agar Scientific) and pretreated with 1% aqueous Alcian Blue 8GX for 10 min before washing in ultrapure MilliQ water. Samples were examined on a Jeol JEM‐1200 Ex II Electron Microscopy and digital images were recorded using the AMT Digital camera (Advanced Microscopy Techniques Corp. 3 Electronics Ave., Danvers, MA 01923 USA, supplied by Deben UK limited, IP30 9QS).

### 2.16 Labelling of lEVs with R18 (Octadecyl Rhodamine B Chloride (R18)) and antibody

lEVs were labelled by adding 1.4 mM of R18 (Thermo Fisher) (made up in an ethanolic solution; < 1% v/v) up to 1 mL. After 1 h at RT in the dark, unincorporated R18 was removed from R18‐lEVs by Size Exclusion Chromatography SEC (using Sephadex G‐75) equilibrated with Hepes/NaCl buffer. The R18: lEVs ratio was estimated from fluorescence measurement before and after removal of unincorporated R18. For labelling with antibody (e.g., anti‐CD63) 1 × 10^9^ lEVs were incubated for 1 h at 37°C with 0.5 µg of anti‐CD63 phycoerythrin (PE) conjugate in 50 µL of 0.22 µm pore size filtered PBS. Unbound antibody was removed by SEC (iZON qEV) according to the manufacturer's instructions. EV containing fractions were pooled and analysed by flow cytometry.

### 2.17 R18 dequenching (lipid mixing) assay

Two assays were performed using R18:

- Time scan dequenching/fusion assay: Here, 1 × 10^5^ R18‐lEVs were allowed to produce a stabilised fluorescence by incubating for 5 min. Using a temperature controlled single cuvette holder (0.5 mL), fluorescence was recorded at 37°C with magnetic stirring on a Hitachi F‐4500 Fluorescence Spectrophotometer (prepared with excitation at 560 nm and emission at 590 nm for measuring R18 fluorescence). Metacyclic trypomastigotes (1 × 10^6^) untreated or treated with trypsin (0.25 % (w/v); 5 min; 37°C) were added 1 min later. At the end of the experiment, Triton X‐100 (1% v/v, final concentration) was added to achieve maximum fluorescence, where the probe is infinitely diluted. The level of fusion could then be calculated from $\%\;dequenching=\frac{100x(F-F0)}{Fmax}\;-F$ where F_0_ is the fluorescence of R18‐lEVs before addition of unlabelled parasite; F is the recorded fluorescence after addition of unlabelled parasites and Fmax if the fluorescence after adding Triton X‐100. The autofluorescence was also measured from unlabelled lEV and parasites to subtract from the measured fluorescences.
- Fusion assay: Unlabelled Mtr (1 × 10^6^) were incubated with R18‐lEVs or unlabelled lEVs (1 × 10^5^) (15 min, 37°C). Parasites treated with trypsin as above in (i) were also used. Unbound or unfused lEVs were removed by washing the parasite‐lEVs mixture in PBS (3×). Fluorescence was measured as in (i) over 100 s and percentage fusion calculated from: $\%\;fusion=\frac{100x(F-F0)}{\left(F-Fmax\right)}\;-F$ where F_0_ is the average fluorescence recorded over 100 s of the Mtr before adding R18‐lEVs; F is the average fluorescence over 100 s of parasite interaction with R18‐lEVs and Fmax is the average fluorescence recorded in 100 s after maximum fluorescence (lysis) with Triton X‐100.

### 2.18 Fluorescence microscopy of interacting lEVs and T. cruzi

Metacyclic forms were incubated with R18‐lEVs at 37°C for 10 min (1 × 10^5^ R18‐lEVs and 1 × 10^6^ metacyclic trypomastigotes) followed by washing with PBS (3×). After fixing the cells (4% paraformaldehyde for 15 min), they were washed 5× and mounted on slides with DAPI‐Vectashield mounting medium (Vector Laboratories). Microscopy was carried out using an Olympus IX81 inverted microscope with a monochromatic camera, U‐CMAD3, images then being coloured with Cell^M software.

### 2.19 Cytokine antibody array and ELISA measurements

To measure inflammatory cytokines, lysed lEVs were applied to the cytokine antibody array (R&D systems) or the TGF‐β1, IL‐13 or IFN‐γ ELISA kits (R&D systems), in triplicate, according to the manufacturer's instructions. For the TGF‐β1 ELISA, this involved activation for 10 min with 1 N HCl (20 µL/100 µL sample) and neutralization with 1.2 N NaOH in 0.5 M HEPES (20 µL/100 µL sample). To control for potential bovine TGF‐β1, an FBS control was also measured (RPMI‐1640 supplemented with FBS (5%)). IL‐13 or IFN‐γ ELISA kits (R&D systems), in triplicate, were carried out according to the manufacturer's instructions.

### 2.20 Measurement of intracellular calcium

Essentially this was carried out as described previously (Ansa‐Addo et al., 2010; Cestari et al., 2012). Briefly, cells stimulated with 5 × 10^6^ infectious metacyclic trypomastigotes, or insect stage epimastigotes as control, were labelled with 2 µM Fura 2‐AM (Sigma Aldrich) with stirring and fluorescence was monitored at 505 nm using a spectrophotometer (Hitachi 4500) upon excitation every second at 340 and 380 nm. The concentration of intracellular calcium ([Ca^2+^]_cyt_) was calculated using the equation [Ca^2+^]_cyt _= *K*_d_[(*R*‐*R_min_*)/(*R_max_*‐*R*)] as described before (Ansa‐Addo et al., 2010). Where thapsigargin was added to uninfected cells, (µM, final concentration) they were maintained in nominal Ca^2+^‐free buffer (140 mM NaCl, 4 mM KCl, 10 mM glucose, 10 mM HEPES‐KOH and 1 mM MgCl_2_, pH 7.3–7.4). Cells infected with Mtr were treated either with SOCE inhibitors (all Sigma‐Aldrich), 2‐APB (30 µM), U‐73122 (5 µM), YM‐58483 (1 µM), EGTA (5 mM), BAPTA‐AM (10 µM), or stimulated with sublytic MAC (and 5% NHS as a control).

### 2.21 Statistical analysis

Statistical analysis (unpaired, Student's *t* test and 1‐/2‐way ANOVA with Sidak's post‐test) was performed using GraphPad Prism software, version 5.0 (GraphPad Software, San Diego, CA). Significance values: ns (non‐significant), *p >* 0.05. The following were considered statistically significant differences: * *p ≤* 0.05, ** p ≤ 0.01, *** p ≤ 0.001, and **** *p ≤* 0.0001.

## 3 RESULTS

### 3.1 Dose‐dependent T. cruzi‐mediated lEV biogenesis in epithelial cells occurs through activated calpain and IP_3_/store‐mediated increase in cytoplasmic calcium

**FIGURE 1:**
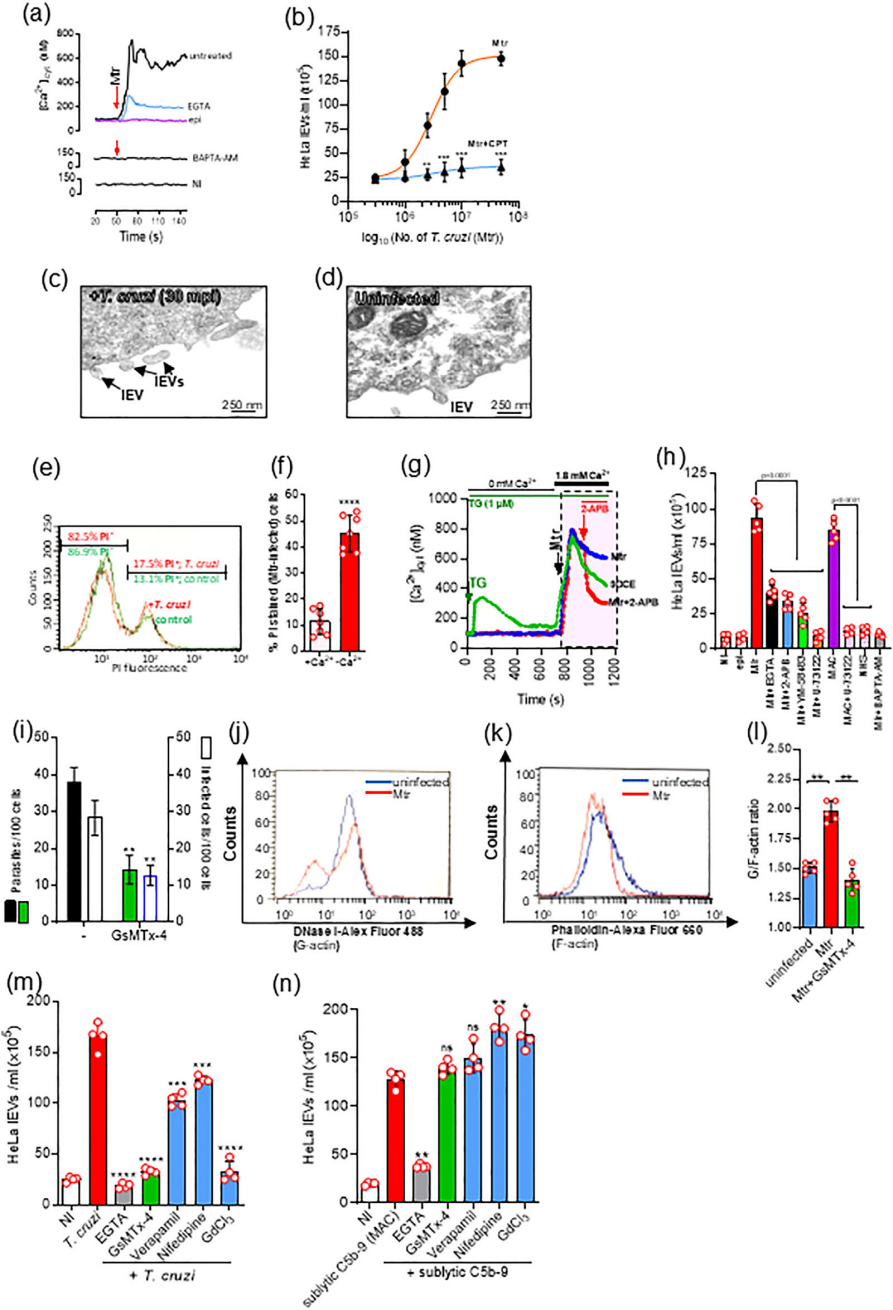
*Trypanosoma cruzi* induces a calcium‐dependent lEV release from epithelial HeLa cells which can be reduced by blocking mechanosensitive surface channels. (a), HeLa cells (1.0 × 10^6^) were loaded with Fura 2‐AM at 37°C in nominal Ca^2+^‐free buffer and for all experiments left to equilibrate by preincubating at 37°C for 5 min, from which point t = 0 s was taken. After 60 s, metacyclic trypomastigotes (Mtr) (5.0 × 10^6^) were added (red arrow) and [Ca^2+^]_cyt_ was monitored using a spectrophotometer over the ensuing 90 s. Mtr infected cells were also treated with EGTA (5 mM) and BAPTA‐AM (10 µM) and epimastigote and non‐infected (NI) controls were included. (b), HeLa cells harvested by trypsinization (1 × 10^6^/well in triplicate) were stimulated at 37°C for 30 min with *T. cruzi* Mtr (5:1, parasites‐to‐cell ratio) also in the presence of calpeptin, CPT (10 µg/mL). The dose‐response curves were fitted with a 4‐parameter logistic equation. Additional analysis used 2‐way ANOVA with Sidak's post‐test. ***p < 0.001, **p < 0.01 (*n *= 6). lEVs released were isolated and analysed as described in Materials and Methods. (c), Transmission electron microscopy of HeLa cell‐derived lEVs being released after stimulation with *T. cruzi* Mtr 5:1 (parasites‐to‐cell) ratio at 37°C for 30 min or of resting, uninfected cells, (d). HeLa cells showing increased injury (%PI^+^), in representative histogram following 30 min exposure of HeLa to metacyclic trypomastigotes (Mtr) (red curves), compared to uninfected cells (green curves), in the presence of propidium iodide, (e). In (f), HeLa cells were injured by a 5:1 ratio of *T. cruzi* metacyclic forms (30 min/37°C) in the presence or absence of Ca^2+^. (g), HeLa cells (1.0 × 10^6^) were loaded with Fura 2‐AM at 37°C in nominal Ca^2+^‐free buffer and left to equilibrate for 5 min, from which point t = 0 s was taken. After 60 s, in calcium‐free buffer, thapsigargin (TG) was added and [Ca^2+^]_cyt_ monitored using a spectrophotometer over the ensuing 600 s, whereupon cells were incubated in 1.8 mM Ca^2+^ to stimulate SOCE (green line). HeLa were also infected with Mtr, (with no TG) but in the presence of extracellular Ca^2+^ (1.8 mM) (blue line). Also in the absence of TG, cells were also infected and then 240 s later, in the presence of Ca^2+^, treated with 2‐APB (5 µM) (red line). In (h), the effect on lEV release from infected HeLa cells of removing extracellular Ca^2+^ with EGTA was monitored. Other treatments included 2‐APB (SOCE modulator), YM‐58483 (CRAC channel inhibitor), U‐73122 (PLC inhibitor) and BAPTA‐AM (cell‐permeant Ca^2+^ chelator). HeLa cells were also stimulated with sublytic MAC (C5b‐9) to release lEVs. HeLa cells (1 × 10^5^/well) when pretreated with the Stretch‐Activated Calcium TRPC blocker, GsMTx‐4, showed marked reduction in infection with Mtr. HeLa cells (1 × 10^6^/well were infected with Mtr and showed reduced invasion with GsMTx‐4 (i)). To measure the G‐actin to F‐actin ratios in uninfected versus *T. cruzi*‐infected HeLa, G‐actin was measured by flow cytometry with DNase 1‐Alexa Fluor 488 conjugate (j) and F‐actin by Phalloidin Alex Fluor 660 (k) as described in Materials and Methods. Results in (l) show mean ± S.D from a representative experiment performed in triplicate, for the G/F‐actin ratios of HeLa cells infected (60 mpi), with or without GsMTx‐4. Upon infection of HeLa cells (1 × 10^6^/well) with Mtr (m), the greatest reduction in host cell mEV release was for GdCl_3_ and GsMTx‐4. EGTA (5 mM) was used as a negative control for parasite‐stimulated Ca^2+^‐mediated mEV release. The inhibitors used to limit T. cruzi‐mediated lEV release did not work when the stimulus for lEV release was sublytic complement (n). Data represents the mean ± SD of three independent experiments performed in triplicate. *p < 0.05, **p < 0.01, and ***p < 0.001 were considered statistically significant.

We showed previously, that infective *T. cruzi* MTr, in contrast to non‐infective epimastigote forms, induced an increase in [Ca^2+^]_cyt_, and stimulated lEV production from THP‐1 monocyte cells (Cestari et al., 2012). Using the Silvio X10/6 strain of *T. cruzi*, previously shown to induce a high level of lEVs from a range of cell types (Cestari et al., 2012; Wyllie & Ramirez, 2017), metacyclic (but not epimastigote) forms, induced in HeLa cells an increase of [Ca^2+^]_cyt_ from 98 nM to 755 nM in 60 s (Figure 1a), which was reduced when extracellular Ca^2+^ was chelated with EGTA. Furthermore, the Mtr‐mediated host cell lEV release was dose‐dependent (Figure 1b) and Ca^2+^‐dependent (Figure 1b, blue line and Figure S2A, purple/green bars). Interestingly, *Salmonella enterica serovar typhimurium* (ST), which also causes a Ca^2+^‐mediated depolymerisation of the actin cytoskeleton (Zhou et al., 1999), stimulated a release of lEVs from HeLa cells (Figure S2A). However, this was not the case with the non‐invasive strain SL1344 mutant (ΔSPI1), (nor with the extracellular parasite *Giardia intestinalis* (Figure S2B)) suggesting that the non‐invasive pathogens tested did not stimulate host cell lEV release, whilst the invasive, intracellular forms, did.

The host lEVs released from Mtr infection of HeLa cells ranged from 0.1 to 0.6 µm in diameter as observed by transmission electron microscopy (TEM) (Figure 1c) and nanosight tracking analysis (NTA) (Figure S2C) indicating a peak modal diameter of 233 ± 28.7 nm (TEM image, inset). The lEVs were positive for EV markers TSG101, ANXA1 (Figure S2D) and exposed PtdSer (Ansa‐Addo et al., 2010), detected by annexin V‐FITC binding (Figure S2E) and were largely positive for CD44 (or H‐CAM, Homing Cell Adhesion Molecule) (Figure S2F), a surface receptor for hyaluronate (Mathieu et al., 2021) indicating that the majority of lEVs were not of parasite origin. Infected compared to uninfected HeLa cells showed increased surface expression of Lamp2 by flow cytometry (Figure S2G) as did the released lEVs (lEVs (30 µg) probed with anti‐Lamp2 by western blotting) (S2H), which also carry TLR4. Exosomes, released when multivesicular bodies (MVBs) fuse with the PM (Figure S2L), are also secreted from cells in a Ca^2+^‐dependent manner, isolation of exosomes (which fall within the size range of sEVs) requiring the supernatant from the 15,000 × *g* lEV pellet to be further centrifuged (100,000 × *g*; 2 h). sEVs were of peak modal size 95 ± 17.5 nm (S2I) and were positive for TSG101 and ANXA1 (S2J). The isolated TSG101^+^ vesicle fractions following sucrose density gradient centrifugation, indicated a buoyant density of 1.10–1.14 g/mL (Figure S2K), and showed the typical “cup‐shaped” morphology, in TEM (Figure S4K, inset). Of the constitutively released EVs from unstimulated HeLa cells (green bars in Figure S2M), the majority (80.2%) were sEVs (Figure S2N and O) but upon infection with parasites (orange bars in Figure S2N and O), the majority (65.1%) were lEVs.

Metacyclic trypomastigotes stimulate store‐operated calcium entry (SOCE) by activating phosphoinositide‐specific phospholipase C (PLC) through gp82/Lamp2 interaction, which stimulates IP_3_‐mediated Ca^2+^ release from ER (Onofre et al., 2021). This store depletion results in Ca^2+^ entry through Orai PM channels and the TRPC (C‐type transient receptor potential) class of ion channels (Lopez et al., 2020). [Ca^2+^]_cyt_ can also increase through receptor‐operated calcium entry, RORE, using PM channels such as P2X (and TRPC) (Itagaki et al., 2005; Protasi et al., 2021) as well as through micro‐injuries to the host PM, so‐called “store‐independent calcium entry” (SICE) (Chamlali et al., 2021). That some microinjuries to the host cell PM are being brought about by Mtr, is apparent from the increased nuclear staining with propidium iodide upon infection (Figure 1e). As expected, this triggers the Mtr‐mediated repair mechanism which only functions in the presence of calcium (Figure 1f).

To establish the relative contribution to lEV biogenesis of increases in [Ca^2+^]_cyt_ from SOCE or SICE, the membrane permeable SOCE modulator, 2‐aminoethyl diphenylborinate (2‐APB) and Ca^2+^ chelator, EGTA, were utilised. To measure the level of SOCE attainable, thapsigargin (TG) was added to uninfected cells (green line in Figure 1g). TG inhibits sarcoplasmic/endoplasmic reticulum Ca^2+^‐ATPase (SERCA) sequestration of cytosolic Ca^2+^ into the ER bringing about a Ca^2+^ leak into the cytosol and thereby passively depleting the ER of its Ca^2+^ store. The depleted ER Ca^2+^ levels stimulate store‐operated calcium entry (SOCE) which in addition to the Ca^2+^ not being sequestered into the ER, means that [Ca^2+^]_cyt_ rises rapidly. In our experiments, in TG‐treated, uninfected cells, the [Ca^2+^]_cyt_ had reached ∼350 nM within 40 s (Figure 4g, green line). Over the subsequent 10 min this almost returned to basal levels (Figure 1g). These cells, in nominal Ca^2+^‐free buffer, now depleted of ER Ca^2+^ stores, were then exposed to 1.8 mM Ca^2+^ to stimulate SOCE (green line). HeLa cells exposed to Mtr alone showed a similar increase in [Ca^2+^]_cyt_, (blue line, Figure 1g). When 2‐APB was added (at the 900 s mark) to Mtr‐exposed HeLa, [Ca^2+^]_cyt_ fell sharply (red line, Figure 1g).

Considering lEV release from HeLa cells exposed to Mtr, where extracellular Ca^2+^ had been chelated with EGTA (black bar, Figure 1h), only Ca^2+^ from intracellular stores (mainly ER) was available for stimulating lEV release and as such no subsequent SOCE (or SICE) could be activated. Compared to untreated, infected cells (red bar), addition of EGTA to infected cells reduced host cell lEV release by 57%. When SOCE was specifically inhibited, with 2‐APB (blue bar, in Figure 1h), which modulates PM channels including Orai1 and TRPC6, the level of lEV inhibition, was similar at 64%. Full lEV biogenesis is therefore dependent on intracellular stores (mainly IP_3_‐mediated Ca^2+^ release from ER) and subsequent SOCE. When SOCE is inhibited with 2‐APB, SICE could still occur, but as EGTA (where neither SOCE nor SICE could occur) conferred no additional inhibition of lEV release compared to 2‐APB, SICE must play little or no role in vesiculation. Due to questions of 2‐APB's specificity (Slowik et al., 2023), a more selective inhibitor of SOCE, YM‐58483, which blocks PM Ca^2+^ channels following depletion of the ER Ca^2+^ store (Ishikawa et al., 2003) and U‐73122 (Peppiatt et al., 2004) which blocks PLC‐mediated IP_3_ production and therefore Ca^2+^ release from the ER, were used. Whilst YM‐58423 limited Mtr‐mediated lEV release to a similar extent (green bar) (73%) as 2‐APB (blue bar), U‐73122 (orange bar), a PLC inhibitor therefore blocking IP_3_‐mediated Ca^2+^ release from the ER and SOCE, completely reduced lEV release to constitutive levels as are released from uninfected cells.

In further support for IP_3_‐mediated [Ca^2+^] increase and SOCE as instrumental in Mtr‐mediated lEV biogenesis, lEV release was stimulated by sublytic complement deposition (membrane attack complex or “MAC”) on HeLa, (purple bar, Figure 1h). Sublytic MAC causes increased [Ca^2+^]_cyt_ not just directly from influx from the extracellular environment, but also partly through release of Ca^2+^ from the ER via IP_3_R channels (Triantafilou et al., 2013), so‐called calcium‐induced calcium release (CICR) (Roderick et al., 2003). As increased [Ca^2+^]_cyt_ can activate PLC to produce the secondary messengers, IP_3_ and DAG (Bagley et al., 2004), which can activate TRPC channels, such as TRPC6/7 (Hofmann et al., 1999) in the PM and stimulate Ca^2+^ influx, we were not surprised to also see U‐73122 abrogate lEV release stimulated by sublytic MAC (pink bar, Figure 1h).

### 3.2 Mechanosensitive calcium channel inhibitor, GsMTx‐4, inhibits parasite‐mediated actin depolymerization, cellular invasion, and host cell lEV release

Many pathogens promote their uptake through interactions of their surface proteins with cellular receptors. For *T. cruzi*, surface glycoproteins such as gp82 and Tc85, stimulate increased [Ca^2+^]_cyt_ by SOCE, leading to increased cellular invasion (Rodrigues et al., 2019). That second messengers DAG and IP_3_ are generated and besides activating SOCE, stimulate receptor‐operated calcium entry (ROCE) by activating Transient receptor potential cation channel, subfamily C (TRPC) channels, prompted us to look specifically at the effect of TRPC inhibitors not just on invasion and actin remodelling but also lEV release. To investigate other *T. cruzi*: host cell interactions and associated signalling pathways that might play an early role in invasion and stimulate lEV release from host cells, we focused on the role of lipid rafts and integrins, since these signalling pathways activated by the parasite, lead to Ca^2+^ mobilisation from intracellular compartments.

Focusing on mechanosenstive channels (MSC) or stretch‐activated channels (SAC), we showed for the first time that treating cells with GsMTx‐4, reduced *T. cruzi* invasion (Figure 1i). GsMTx‐4 is a peptide derived from tarantula toxin, which by disturbing the lipid‐channel boundary, is a potent inhibitor of cationic mechanosensitive channels (MSCs) such as TRPC1, TRPC6, (Spassova et al., 2006) and Piezo channels. Compared to the G/F actin ratio for uninfected HeLa, 1.5, (Figure 1j, k, and l), raised upon infection to 1.98 (Figure 1l), this was reduced with GsMTx‐4 (to 1.4) indicating a reduction in depolymerization of actin (reduced conversion of F‐ to G‐actin). The increased depolymerization induced by Mtr relates to the increased cellular invasion as noted previously (Cortez et al., 2006; Ferreira et al., 2006) and explains why actin depolymerization and invasion is reduced by GsMTx‐4. In decreasing actin depolymerization this also accounts for the decrease in lEV release (Figure 1m). We also found reduced parasite‐induced host cell lEV release (Figure 1m), with the L‐type calcium channel blockers, verapamil and nifedipine and the MSC inhibitor, gadolinium chloride (GdCl_3_), but not when cells were stimulated with sublytic complement (Figure 1n).

Integrin binding induced by raft clustering is proposed to modulate cytoskeleton rearrangement (Bodin et al., 2005). We also confirmed that *T. cruzi* metacyclic entry was reduced upon pretreatment of HeLa cells with RGD (an inhibitor of integrin‐ligand interactions), in a dose‐dependent manner (Figure S3A) but not with the control peptide, RGE, (Figure S3B). lEV release was also reduced with RGD as it was with anti‐CD49d (that binds to integrin α‐chain), most likely by inhibiting parasite‐integrin interaction, so limiting any resulting increase in [Ca^2+^]_cyt_/actin depolymerisation. However, the reduced lEV production only occurred when the stimulus was *T. cruzi* (Figure S3C) and not when it was sublytic complement (Figure S3D) (which also stimulates lEV release (Stratton et al., 2015), confirming that the parasite‐mediated activation of the receptor is required to stimulate lEV release. Furthermore, cholesterol‐depleting, and likely lipid raft‐disrupting MβCD, inhibited *T. cruzi* entry (Figure S3E) and lEV release (Figure S3F), but not cholesterol sequestering filipin, nor nystatin (Figure S3E and F).

In this section we showed that the Mtr‐ and SOCE‐mediated increase in [Ca^2+^]_cyt_, and cellular invasion is accompanied by increased actin depolymerization and lEV release, which is specifically inhibited with the TRPC inhibitor, GsMTx‐4.

### 3.3 Reduced invasion in epithelial cells (HeLa) treated with antagonists of lEV biogenesis is not restored by supplementation with HeLa lEVs, whether from uninfected or infected cells

**FIGURE 2:**
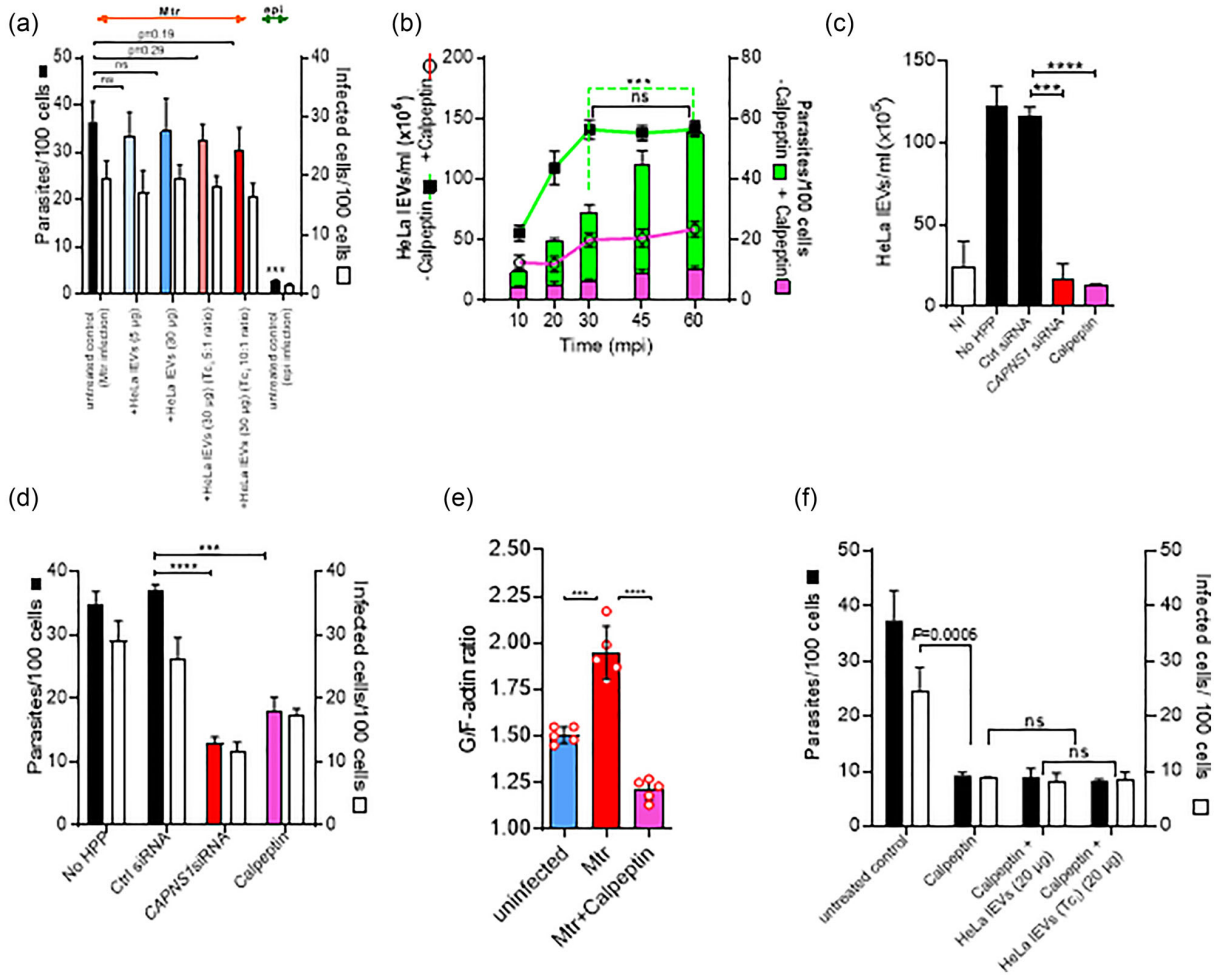
Inhibition of lEV release from host HeLa cells using the actin remodelling drug calpeptin, is not restored by supplementation of host lEVs, whether from infected cells or not. (a), There is no increase of cellular invasion of HeLa cells with Mtr, whether HeLa lEVs are added from infected or uninfected cells. (b), Kinetic analysis of lEV release (lines) and T. cruzi metacyclic invasion (bars) before and after pretreatment (37°C for 30 min) with calpeptin (20 µM). Without treatment (green line), T. cruzi‐elicited release of lEVs increased over the first 30 min, but parasite invasion (green bars) continued beyond this point. However, calpeptin inhibited lEV release (pink line) and abrogated parasite entry (pink bars). Calpeptin and CAPNS1 siRNA block lEV release (c) and inhibit invasion (d). The G/F‐actin ratios of calpeptin‐treated, Mtr‐infected HeLa cells, 60 mpi, are significantly reduced, (e). Addition of lEVs (10^7^/mL) from infected or uninfected HeLa did not restore invasion levels reduced by calpeptin, (f).

HeLa lEVs, from uninfected cells, added to untreated HeLa cells, had no effect on invasion levels, even when added in excess, (12‐fold higher concentration than in conditioned medium of Mtr‐infected cells; blue bars, Figure 2a)), implying no function as decoys to infecting Mtr. Even addition of 30 µg lEVs from HeLa cells infected with Mtr (5:1 or 10:1 parasite: host cell ratio) reduced infection non‐significantly by only 8.6% and 12.8%, respectively (orange bars, Figure 2a). However, to establish whether lEVs from infected cells can act as true decoys will require further investigation. Even though following infection, the cells had increased surface expression of Lamp2 (Figure S2G), as did the released lEVs (Figure S2H) implying a better capability of acting as decoys by competing for Mtr (gp82) / HeLa cell (Lamp2) interactions, set against this there is the many‐fold size differential between lEVs (200–500 nm) and Mtr (16–42 µm). TLR4 which can also bind the Mtr‐specific gp82 was also detected on the lEVs from infected HeLa (Figure S2H). We then investigated the process that modulates actin remodelling and lEV release during infection of cells to better understand its role(s) in cellular infection. It is well documented that calpain, a cysteine protease, has several functions during the production of lEVs. These include hydrolysis of actin‐binding proteins such as talin, α‐actinin, spectrin, filamin and the ERM (ezrin/radixin/moesin) protein, ezrin, resulting in cleavage of constituents of stress fibres (actin filament bundles) (Pollard, 2016). This leads to cytoskeletal rearrangements which facilitate shedding of lEVs (Tricarico et al., 2017).

Kinetic analysis of *T. cruzi* invasion in cells showed that although it accompanied increasing cellular release of lEVs up to 30 min post infection (30 mpi) (Figure 2b—green bars and green line, respectively), between 30 to 60 mpi, the increasing invasion did not accompany host lEV production: lEV release stopped increasing between 30 and 60 mpi, whilst invasion continued to increase. Both lEV release and invasion were significantly reduced in the presence of calpeptin, a specific calpain inhibitor (Bassé et al., 1994) (Figure 2b, pink line and pink bars, respectively). In further confirmatory experiments, to exclude any possible non‐specific effects of calpeptin, we carried out a knockdown of both µ‐ and m‐calpain isoforms using a calpain small‐subunit 1 small interfering RNA (*CAPNS1* siRNA). Effectiveness of silencing (using siRNA#6) was confirmed by the lack of expression of CAPNS1 (western blotting in Figure S4A), by flow cytometry analysis (Figure S4B and S4C) and immunofluorescence microscopy (lack of punctate fluorescence in Figure S4D (1) versus (2) and (3)). Silencing of *CAPNS1*, as with calpeptin, significantly inhibited *T. cruzi*‐mediated lEV release (Figure 2c) and reduced parasite invasion of HeLa cells (Figure 2d). Increased actin depolymerization upon infection was reduced with calpeptin (Figure 2e) and unsurprisingly infection levels were not restored to calpeptin‐treated cells in the presence of separately harvested and added lEVs (20 µg lEVs), whether from uninfected or infected HeLa cells (Figure 2f).

### 3.4 THP‐1 monocyte lEVs undergo a fusogenic interaction with T. cruzi metacyclic trypomastigotes

**FIGURE 3:**
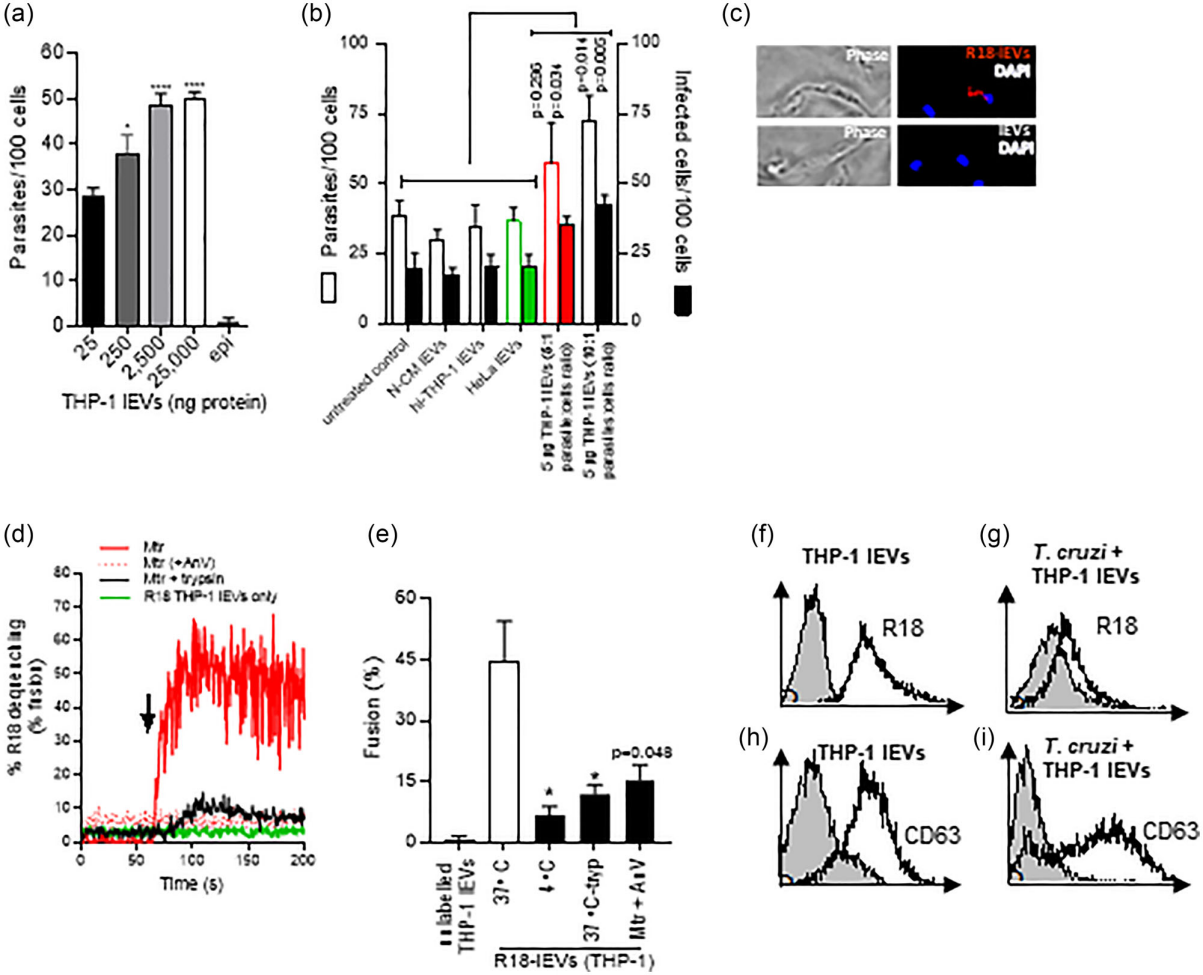
lEVs from THP‐1 monocytes carrying latent TGF‐β1 fuse with Mtr, activated TGF‐β1 being released to enhance cellular invasion. (a), Mtr infection of HeLa increases dose dependently with increasing added THP‐1 lEVs. (b), invasion assays in which HeLa were treated with 5 µg THP‐1 lEVs (5:1 and 10:1 parasite:cell ratio). N‐CM (non‐conditioned medium); hi‐THP‐1 lEVs (heat‐inactivated lEVs). (c), *T. cruzi* Mtr were incubated with R18‐labelled and unlabelled THP‐1 lEVs as described in Materials and Methods and observed by fluorescence microscopy after fixation and mounting with DAPI‐Vectashield. (d), Using the time scan R18 dequenching assay, unlabelled *T. cruzi* Mtr or trypsinized Mtr (10^6^) (0.25% trypsin/5 min/37°C) were incubated after 60 s (indicated by arrow) with R18‐labelled THP‐1 lEVs (2.5 µg). As control, R18‐lEVs were incubated without Mtr (green line). Fluorescence readings were obtained (excitation/emission 560 nm/590 nm) for the period prior to and then for the 100 s after addition of R‐18 lEVs to give a % dequenching over time. Maximum fluorescence was obtained by adding Triton X‐100 (1% v/v). (e), Percentage fusion was calculated using the formula in Materials and Methods and showed that fusion was temperature and lEV surface protein‐dependent (lEVs treated with 0.25% trypsin (10 min/37°C)) as well as dependent on Mtr‐PtdSer. (f and g), shows acquisition of R18‐ and in (h) and (i) of anti‐CD63‐labelled lEVs to the surface of Mtr by flow cytometry. Unlabelled lEVs are represented by filled histograms in (f) and (h) and *T. cruzi* with added unlabelled lEVs shown in (g) and (i).

We previously observed increased cellular invasion by *T. cruzi* in the presence of THP‐1 monocyte lEVs (Cestari et al., 2012), and showed these lEVs to protect the parasite against complement attack (Cestari et al., 2012). Here, we first confirmed this enhanced invasion with increasing amounts of THP‐1‐derived lEVs interacting with Mtr (Figure 3a and b). To test the nature of the interaction between monocyte lEVs and parasite, we labelled the lEVs with octadecylrhodamine (R18). Fluorescence microscopy showed possible attachment to parasite (Figure 3c). To test the fusogenic capacity of the lEVs we carried out an R18 dequenching assay to measure fusion kinetics. The R18‐lEVs were combined with unlabelled Mtr forms at a ratio of 1:10. As a control, R18 labelled lEVs alone were used and showed negligible R18 dequenching (green line in Figure 3d). Within 25 s of addition of unlabelled Mtr forms to R18‐lEVs, there was ∼ 50% dequenching. Suggesting that any possible fusion (lipid mixing), or possibly only “hemifusion,” may be mediated by prior interaction of parasite/lEV proteins, Mtr treated with trypsin to remove surface proteins, showed no evidence of fusion (black line in Figure 3d). When these fusion assays were conducted at 4°C, or where exposed phosphatidylserine (PtdSer), which is expressed on Mtr (Damatta et al., 2007), was blocked with AnV, no fusion was observed (Figure 3d and e). We also showed R18 and CD63 labelling of *T. cruzi* after incubating with R18‐labelled and unlabelled lEVs, by flow cytometry (Figure 3f‐i).

### 3.5 Enhanced, TGF‐β1‐mediated, cellular uptake of T. cruzi, occurs through parasite activation of latent TGF‐β1 on monocyte lEVs

**FIGURE 4:**
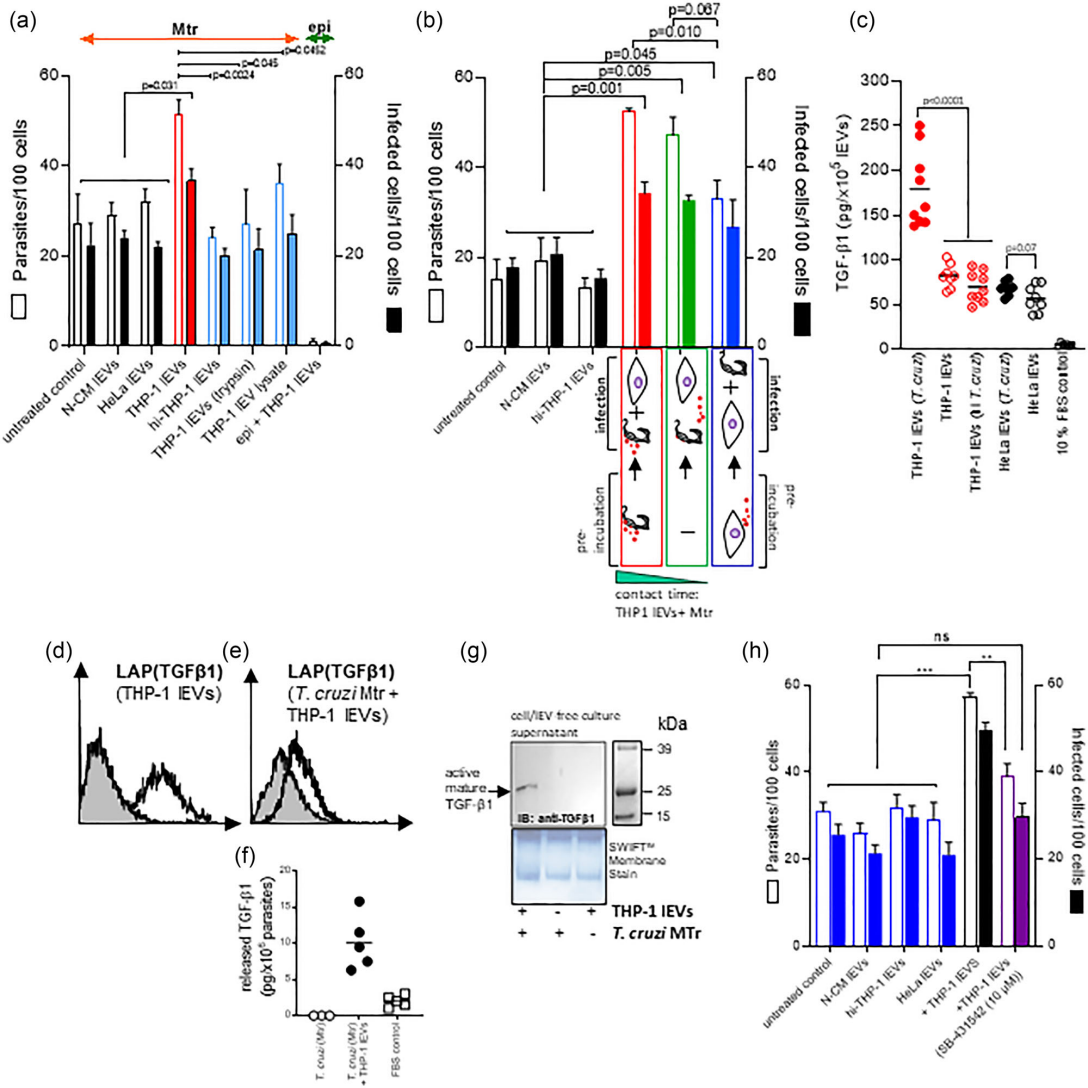
THP‐1 monocyte lEVs carrying latent TGF‐β1 release activated TGF‐β1 upon fusion with Mtr to enhance cellular invasion. In (a), cellular infection levels are shown with added intact THP‐1 monocyte lEVs, and as control: lEVs from non‐conditioned medium (N‐CM), heat inactivated lEVs, trypsin‐treated lEVS, and a lEV lysate (0.1% Triton X‐100 + protease inhibitor cocktail); infection with epimastigotes was also used as control. (b), Infections were carried out in which either Mtr were preincubated with THP‐1 lEVs (red bars), or in which there was no preincubation (green bars), and in which HeLa cells to be infected were incubated with THP‐1 lEVs, which were then removed at the point of infection (blue bars). (c), ELISA measurements of activated TGF‐β1 (10 min with 1N HCl, as per Materials and Methods) for lysed lEVs from HeLa stimulated with *T. cruzi* or *T. cruzi* (heat inactivated) and from lEVs released from untreated HeLa and THP‐1 cells. (d), shows detectable LAP‐TGF‐β1 on the surface of THP‐1 lEVs, also observed on Mtr upon incubation with THP‐1 lEVs, (e), as well as released TGF‐β1 detected by ELISA, (f). (g), western analysis of TGF‐β1 released into the serum‐free cell culture supernatant (rendered cell‐ and lEV‐free by differential centrifugation) and concentrated using a Microcon‐10 kDa Centrifugal Filter Unit with Ultracel‐10 membrane; samples of equal protein concentration were loaded for western analysis using anti‐TGF‐β1. (h), Reduced invasion levels are demonstrated upon addition of lEVs from *T. cruzi*‐stimulated THP‐1 monocytes, when TβRI signalling is blocked with SB‐431542.

Further supporting the importance of protein interaction between lEVs and Mtr, prior to any fusion event, the enhanced invasion in vitro with THP‐1 lEVs was not achieved when the lEVs were heat inactivated or pretreated with trypsin and only partially achieved when lysed lEVs were added (blue bars, Figure 4a). Interestingly, we found that in in vitro invasion assays, the longer the exposure of Mtr to THP‐1 lEVs (red bars compared to green bars, Figure 4b), the greater the level of invasion and that if the lEVs were preincubated with host cells and then removed prior to initiating infection with Mtr (blue bars, Figure 4b), the increase in invasion compared to controls was only just significant (*p* = 0.045).

There are several cytokines upregulated in the lEVs released from THP‐1 monocytes following interaction with *T. cruzi* (Figure S5A and B) with significant fold increases for: IL‐16 (2.35), IL‐32α (2.18), IL‐1α (2.09), Serpin E1 (PAI‐1) (1.94) and IL‐13 (1.51) (Figure S5C), which are all proinflammatory apart from IL‐13. IL‐13 and IFN‐γ were measured at 192 ± 63 pg and 880 ± 497 pg from 5 × 10^5^ lysed lEVs, respectively. We decided to further focus on the role of the anti‐inflammatory cytokine, TGF‐β1, which we had previously detected on THP‐1 lEVs (Cestari et al., 2012). We were also most interested in this cytokine because it is found on the cell surface (and therefore released on shed lEVs) in a membrane‐bound form; this can then be activated and released from its latent complex to bind TGF‐β1 receptors. The level of activated TGF‐β1 (by treating with 1.0 N HCl as described in Materials and Methods) in lysed lEVs from infected THP‐1s was 176.3 ± 41.4 pg/×10^5^ lEVs (Figure 4c), compared to lEVs from uninfected cells at 79 ± 15,1 pg/×10^5^ lEVs. Upon incubation (30 min; 37°C) of THP‐1 lEVs (1 × 10^7^ (2.5 µg)) with *T. cruzi* Mtr forms (1 × 10^6^), TGF‐β1, detectable on THP‐1 lEVs (Figure 4d), was also detectable on the parasite (Figure 4e) by flow cytometry (detected as bound to LAP) and shown by ELISA to be detected (as released TGF‐β1) only after addition of lEVs to parasite (Figure 4f). Next, we investigated the possibility that the parasite activates the TGF‐β1 which is likely trapped in a latent complex on the THP‐1 lEVs that fuse with Mtr, to release the 25 kDa TGF‐β1 homodimer. For this, *T. cruzi* Mtr were treated with THP‐1 lEVs, as before. We were then able to detect the 25 kDa, active, mature TGF‐β1 in the concentrated (using a Microcon‐10 kDa Centrifugal Filter Unit) cell‐ and EV‐free cell culture supernatant (Figure 4g). Finally, in vitro invasion experiments showed that the elevated cellular invasion of HeLa cells with Mtr and added THP‐1 lEVs, could be reduced to normal levels (as without lEV supplementation, non‐conditioned medium or heat‐inactivated lEVs; blue bars in Figure 4h) by blocking TGFβR1 signalling (SMAD2/3 phosphorylation) with SB‐431542, the inhibitor of TGF‐β signalling that *T. cruzi* infection activates (purple bars in Figure 4h).

## 4 DISCUSSION

*T. cruzi* metacyclic trypomastigotes (Mtr) trigger an increase in intracellular free Ca^2+^ transients during their invasion of host cells (Burleigh & Andrews, 1998; Burleigh & Woolsey, 2002; Cestari et al., 2012). Such increases lead to calpain‐mediated reorganisation of the actin cytoskeleton and release of lEVs from the surface membrane (Fox et al., 1990; Rothmeier et al., 2015). lEVs play roles in a wide range of diseases (Inal et al., 2012) and whilst in infectious disease, much attention is paid to pathogen lEVs, the role of lEVs from infected host cells or immune cells during infection has been much less well characterised (Lam et al., 2018; Théry et al., 2018). In this study, we confirmed our previous findings that immune cell derived lEVs facilitate *T. cruzi* Mtr parasite entry (Cestari et al., 2012), and delineated specific parasite‐host cell interactions mediating lEV release. Importantly, the current study also elucidated the mechanism by which monocyte lEVs enhance invasion, through investigating their capacity to deliver TGF‐β1 to the Mtr for activation.

### 4.1 T. cruzi infection of epithelial cells and lEV biogenesis is mediated through calpain, activated from IP_3_/store‐released calcium, and can be modulated by inhibiting mechanotransduction

We had previously found *T. cruzi* MTr, but not epimastigotes, to stimulate lEV release in vitro and in vivo, prior to cellular invasion (Cestari et al., 2012) and now confirmed with intracellular *T. cruzi* MTr forms, but not epimastigotes, the Ca^2+^ dependency of this Mtr‐mediated lEV release from HeLa cells. *T. cruzi* is able to trigger calcium signalling in host cells through engaging host receptors (Fernandes & Andrews, 2012) or by causing microlesions in the host cell surface (Fernandes & Andrews, 2012; Oliveira et al., 2021). In infection with Mtr, gp82 interaction with Lamp2 triggers host cell actin remodelling and further lysosomal recruitment to the PM through induction of the PI3K pathway. Ca^2+^ is also released from the ER via the stimulation with IP_3_ of the IP_3_R. Depletion of the ER Ca^2+^ store, is detected by stromal interaction molecules (STIMs), Ca^2+^ sensors in the ER, triggering SOCE through Ca^2+^ release activated channels (CRACs) that include Orai channels and TRPC channels at the PM (Ambudkar et al., 2017). Cytoplasmic Ca^2+^ is then sequestered into the ER through SERCA‐ATPase. Showing the importance of SOCE in *T. cruzi*‐mediated lEV release, we found a 57%–64% decrease in lEV release following infection with Mtr, where cells had either no access to extracellular Ca^2+^ and therefore uptake through SOCE or where extracellular Ca^2+^ was present but SOCE specifically inhibited. This was compared to untreated, control infections where ER Ca^2+^ was available through IP_3_/IP_3_R and SOCE, as well as any non‐CRAC PM channels.

Metacyclic trypomastigotes can stimulate membrane repair mechanisms through perturbations to the host cell PM mediated by the Mtr flagellum. The use of such mechanical stress may induce a breach in the PM and activate MSCs. It was also interesting to demonstrate, therefore, abrogation of host cell lEV release and associated reduced invasion, using MSC inhibitors, GdCl_3_, and with the TRPC inhibitor, GsMTX‐4 (Bowman et al., 2007). GsMTX‐4 perturbs the lipid‐channel boundary and blocks the TRPC channels, such as TRPC1 or TRPC6, whether activated by diacylglycerol (chemical lipid sensing) or stretch (mechanical sensing); TRPC6 for example is thus also a sensor of membrane stretch, whether osmotically or mechanically induced (Spassova et al., 2006). Inhibition of TRPC with GsMTx‐4 which blocks Ca^2+^ influx (Spassova et al., 2006) also brought about a reduced Mtr‐mediated depolymerisation of β‐actin.

We demonstrated *T. cruzi*’s induction of lEV release, and subsequent increased invasion, to occur through activation of several receptors/domains, including lipid raft microdomains and integrins. Abrogation of these signalling routes with MβCD and RGD peptide, respectively, inhibited lEV production, and reduced parasite entry into cells. During *T. cruzi* invasion, interaction of RGD‐containing proteins with host cell integrins increases [Ca^2+^]_cyt_ causing actin depolymerisation. Invasion of macrophages can be blocked with anti‐integrin antibodies (Fernandes et al., 2007), such as anti‐β1 or to a lesser degree with anti‐α4. RGD‐containing peptides, which block integrin‐ligand interaction have also been used to reduce trypomastigote invasion of cardiomyocytes (Calvet et al., 2004), probably by competing with RGD‐containing parasite proteins such as dispersed gene family protein 1 (DGF‐1) (Kawashita et al., 2009) or *Leishmania major* secreted protein gene 1 (Lmsp1) (Campos‐Neto et al., 2003). Since RGD peptide blocks integrin‐ligand interaction, which can activate latent TGF‐β1 (Dong et al., 2017; Shi et al., 2011), it could also be used to ascertain whether epithelial cells themselves, overexpressing integrins such as αvβ6 (Ahmed et al., 2002), could directly activate TGF‐β1 on lEVs. There is a precedent for epithelial cell integrins directly activating latent TGF‐β1 on lEVs, as we found pre‐incubation of HeLa cells with THP‐1 lEVs, subsequently removed prior to infection, to slightly increase cellular invasion (Figure 4b). As a control to demonstrate the specificity of the various inhibitors of parasite‐stimulated lEV release, we used the deposition of complement (sublytic membrane attack complex, MAC, or C5b‐9), which also increases [Ca^2+^]_cyt_, stimulating lEV release, but without activating the same signalling pathways. Looking ahead, by using *T. cruzi* strains representative of the major genotypes of the parasite, taken from the Discrete Typing Units, lEV biogenesis could also be studied to delineate specific virulence factors and their possible role in stimulating lEV release from host cells and in promoting infection.

### 4.2 Host lEVs do not act as decoys to cellular infection with T. cruzi, and reduced invasion upon inhibition of actin depolymerization with calpeptin, is not restored with added EVs

We recently commented on the role of calpeptin in controlling viral infection through its inhibition of lEV release from host cells (Inal et al., 2022; De Sousa et al., 2022). In the current study, we found that inhibiting lEV release with calpeptin or with CAPNS1 siRNA, which silenced calpain subunits 1 and 2, significantly reduced *T. cruzi* invasion. In other work, using pantethine‐treated mice (Penet et al., 2008) or *ABCA1* ^−/−^ mice (Combes et al., 2005), both presented with reduced plasma lEV (“microparticle” (MP)) levels. In both conditions there was complete resistance to the development of cerebral malaria by *Plasmodium berghei* ANKA infected mice (Combes et al., 2005; Penet et al., 2008). It was later shown that adoptive transfer of these plasma MPs from inflamed vessels, caused breakdown of the blood brain barrier, simulating a similar pathology to that in the mouse model of cerebral malaria (El‐Assaad et al., 2014). Generally, host lEV levels are raised in infectious disease (Coakley et al., 2015; Hind et al., 2010), as for example in the plasma of patients with malaria (Antwi‐Baffour et al., 2020; Combes et al., 2004), and HIV (Hubert et al., 2015), and in certain cases lEVs have been shown to be involved in parasite cytoadherence (Evans‐Osses et al., 2017; Faille et al., 2009). Activation of calpains through binding of Ca^2+^ can activate several cellular processes (Catalano & O'Driscoll, 2020). These include: (1) modulation of inflammation (through calpain‐mediated activation and release of matrix metalloproteinase proteinases (MMPs) in turn regulating chemokine activity) (Ji et al., 2016) or increasing cancer cell invasion (also due to calpain's activation and release of MMPs) (Chen et al., 2019) and (2) increase in cell migration (due to calpain‐mediated proteolysis of talin and FAK) (Kerstein et al., 2017). Of relevance to the process of Mtr infection studied here, activated calpain can also bring about: (3) an increase in cellular infection with Mtr (due to calpain‐mediated increase in actin depolymerization) especially in chronic Chagas cardiomyopathy where MMP‐2 and MMP‐9 (which are activated by calpains) play a role in cardiac remodelling (Baron et al., 2022) and (4) an increase in lEV release (due to the action of calpain on cortactin and other cytoskeletal proteins) (Taylor et al., 2020). lEV release is the downstream consequence of calpain‐mediated action on cortactin. Therefore, following the inhibition of calpain‐mediated lEV release in HeLa cells, where there are reduced invasion levels, the cellular infection would not be recoverable by mere restoration of lEV levels.

During infection with Mtr, lEV release was reduced by calpeptin, the G:F‐actin ratio falling to below the 1.5 found for uninfected cells. As already described by others (McNeil & Kirchhausen, 2005), reduced invasion occurs alongside impaired depolymerisation of actin (a G:F‐actin ratio reduced to below uninfected, control levels (< 1.5)). We measured a depolymerization of F‐actin to G‐actin (increased G:F actin ratio) in HeLa cells infected with *T. cruzi* Mtr Sylvio X10/6 at 1 hpi. Working with Mtr infection of fibroblasts, at 72 hpi, actin depolymerization was also noted this time measured by densitometry of western blots (decreased F:G actin ratio). However, as has been described in numerous studies (Bonifácio et al., 2022; Ebstrup et al., 2021; Ferreira et al., 2021; Mott et al., 2009; Rodríguez‐Bejarano et al., 2021; Woolsey & Burleigh, 2004), actin remodelling is a very dynamic process during intracellular invasion with both actin polymerization and depolymerization occurring at different times during cell entry and much later for egress, and sometimes at specific locations within the cell. In the early stages, polymerization of actin microfilaments is required to extend pseudopodia for macropinocytosis/phagocytosis. With regard PM repair, lysosomal exocytosis requires different states of actin polymerization for transport, then vesicle fusion with the PM followed by endocytosis of the damaged membrane. PM shedding of host cell MVs (ectocytosis), involved in membrane repair of the PM, possibly stimulated by Mtr:host cell interaction, also requires local actin depolymerization. Even filopodia whose formation is driven by F‐actin may then require actin depolymerization to release MVs from their surface. At later time points in the infection cycle, actin polymerization is needed to prevent exit from the cell.

### 4.3 Host monocyte lEVs interact with metacyclic trypomastigotes activating latent TGF‐β1 on monocyte lEVs and increasing invasion

Since the main leukocyte types that trypomastigotes infect at the site of infection are monocytes and macrophages (Padilla et al., 2023), we investigated the effect of THP‐1 monocyte‐derived lEVs on the cell invasion process. In contrast to the negligible effect on invasion of epithelial cell lEVs (HeLa), those from innate immune cells, infected with Mtr, showed enhanced uptake. THP‐1 lEVs also showed evidence of membrane fusion with Mtr which we characterised using R18‐labelled lEVs (R18‐lEVs) by immunofluorescence microscopy and dye dequenching. As *T. cruzi* Mtr demonstrate classical apoptotic mimicry, in which PtdSer is exposed (so stimulating parasite uptake and a subsequent anti‐inflammatory response) (Damatta et al., 2007; Wanderley et al., 2020), the host cell‐derived proteins on host lEVs, mediating their adherence to Mtr, are likely to be some of the over 15 PtdSer‐binding proteins so far described (Vorselen, 2022). The variation in expression of these proteins, whether integral or peripheral, in different cells and on their corresponding lEVs, may account for differing abilities to interact with Mtr and to present TGF‐β1. Although putative host lEV:Mtr fusion was detected, this is unlikely to be a prerequisite for TGF‐β1 activation, as lEVs merely “tethered” to the parasite surface (such as through PtdSer: receptor interactions (Vorselen, 2022)) are still likely to have TGF‐β1 activated. Indeed, TGF‐β1 from the host cell being infected or nearby cells could also be activated by the parasite and promote cellular uptake, so acting in an autocrine or paracrine fashion, respectively.

Preincubating Mtr with AnV reduced %R18 dequenching (lipid mixing), further suggesting PtdSer to play a role in lEV binding/fusion to Mtr. It is also likely that a myeloid cell‐derived lEV has more PtdSer receptors and other proteins that can bind PtdSer on metacyclic forms, than would be found on an epithelial cell‐derived lEV. Although TGF‐β1 is more highly expressed on lEVs from infected THP‐1 monoyctes than on lEVs from infected HeLa epithelial cells, the higher invasion levels with THP‐1 lEVs, therefore, may be also related to relative avidity of interaction with Mtr. This would be because of the increased PtdSer receptor expression on monocyte lEVs (and chance for a multivalent interaction with exposed PtdSer on Mtr) compared to the more limited PtdSer receptor expression on epithelial cell lEVs. Previous examples of EV membrane: cell fusion have included glioma cells transferring EGFRvIII between cells via microvesicles (Al‐Nedawi et al., 2008) and exosomal transfer of miRNAs between bone marrow‐derived dendritic cells (DCs), the latter as in our study similarly demonstrated by R18 dequenching membrane fusion assay (Montecalvo et al., 2012). Interestingly in both cases, the EV:cell membrane fusion involved myeloid cells (glial or DC); similarly, our fusion experiments involved monocyte lEVs. PtdSer has certainly been suggested as playing a part at least in binding and possibly internalization (Feng et al., 2010; Matsumoto et al., 2017; O'Dea et al., 2020), and so such fusogenic interactions may be more relevant to myeloid cells expressing PtdSer receptors.

From the work of Waghabi et al. (2005), it was shown that TGF‐β, present in the flagellar pocket of *T. cruzi* amastigote forms, could be internalised, such that this host‐derived immunoreactive TGF‐β enabled completion of the parasite cycle (Waghabi et al., 2005). Earlier it was shown that TGF‐β‐mediated stimulation of infection was dependent on a complete TGF‐β signalling pathway and that when *T. cruzi* attached to epithelial cells lacking TGF‐ β receptor I or II, the parasites could neither penetrate or replicate in these cells, from which it was speculated that the parasite itself might trigger activation of the TGF‐β signalling pathway; more recently Waghabi et al. (2007) found that parasite invasion of cardiomyocytes was reduced in the presence of the TGF‐βR1 inhibitor, SB‐431542 and that as well as reducing the number of parasites per cell, reduced the differentiation of trypomastigotes and their release (Hall & Pereira, 2000; Ming et al., 1995; Waghabi et al., 2007). Recently, in an in vitro model using cardiac cells and Mtr Y strain, decreased invasion and of number of parasites per infected cells was reported in the presence of anti‐TGF‐β1 antibodies (Ferreira et al., 2022). In our study, lEVs released from host immune cells following interaction with parasite, promoted invasion of HeLa epithelial cells with *T. cruzi* Silvio X10/6. Essentially, there was activation of TGF‐β1 delivered to *T. cruzi* metacyclic forms by lEVs derived from infected monocytes. A recent study also looking at EVs released from cells (macrophages infected with *T. cruzi* Y strain) as well as EVs released directly from parasites, showed increased gene expression of proinflammatory cytokines (IL‐1β, TNF‐α, and IL‐6) through TLR2 and NF‐κB (Cronemberger‐Andrade et al., 2020). At this early stage, it is not yet clear if these effects of host cell and parasite EVs on invasion are cell‐specific. In our experiments, that enhanced invasion could be due to TGF‐β1 delivered by the THP‐1 lEVs is a possibility as infection was reduced by blocking TβRI‐mediated signalling with SB‐431542. We detected active, mature TGF‐β1 in the concentrated cell‐free and EV‐free cell culture medium, only when THP‐1 lEVs were incubated with Mtr (Figure 4g), suggesting that the Mtr themselves may activate TGF‐β1, thereby releasing the cytokine from its latent complex, delivered by monocyte THP‐1 lEVs. This will require further confirmation but one possibility could be that the latent host cell TGF‐β1 delivered by monocyte lEVs is activated by *T. cruzi*’s cysteine peptidase, cruzipain (or plasmin from urokinase plasminogen activator (uPa‐) catalysed plasminogen), as was described previously for trypomastigote and amastigote forms’ activation of latent TGF‐β1 from cells (Damatta et al., 2007; Ferrão et al., 2015). In other work, monocytes interacting with opsonized *Candida albicans*, through monocyte Complement Receptor 3 (CR3 or CD11b/CD18), released EVs carrying inactive TGF‐β1 (that could be activated intracellularly); similar EVs could also be released upon apoptotic cell interaction with monocytic CR3. This work raised the possibility that the TGF‐β1 on EVs may bind directly with TGFβRII and be activated intracellularly (Halder et al., 2020)). These monocyte EVs were immune inhibitory and enhanced further secretion of TGF‐β1 from endothelial cells.

**FIGURE 5:**
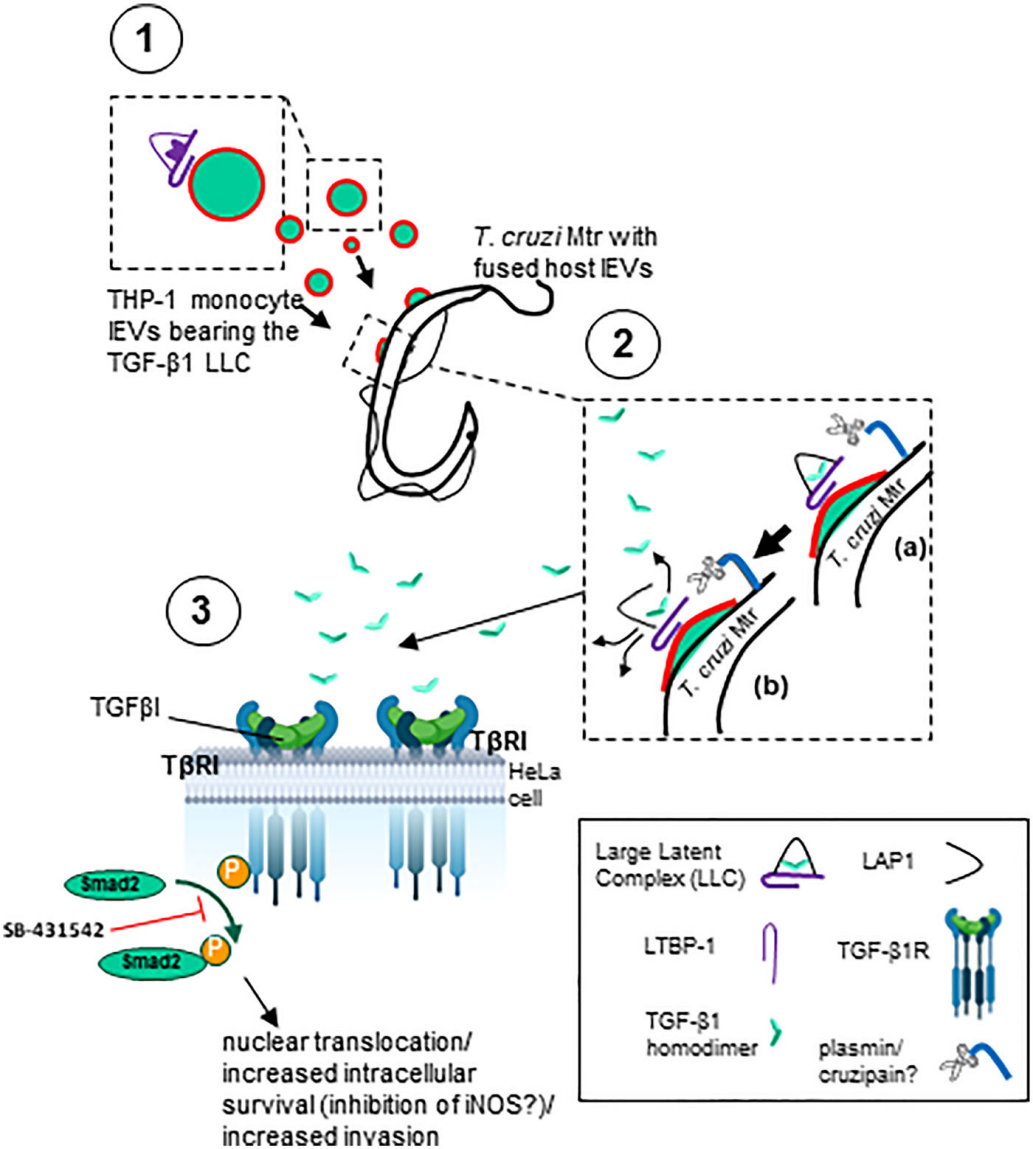
Model for possible activation of host TGF‐β1 from monocyte cells by *T. cruzi* metacyclic trypomastigotes. (1) Monocytes carrying TGF‐β1, within the large latent complex (LLC), a tripartite complex of TGF‐β, LAP, and LTBP‐1 attach to the metacyclic trypomastigotes surface where (2) they are activated by surface proteases such as cruzipain or plasmin (from uPA‐activated plasminogen), releasing the TGF‐β1 homodimer to (3) interact with its cognate TGF‐β1 receptor, TβRI, whereupon Smad2 is activated and phosphorylated and then translocating into the nucleus, resulting in increased survival/invasion.

We report for the first time, therefore, the presentation of latent TGF‐β1 on the surface of lEVs (as opposed to on the surface of host cells) to recipient parasitic cells (Figure 5). The latent TGF‐β1 is on a myeloid cell‐derived lEV lacking an extracellular matrix (Patel et al., 2023) and so is likely attached to the PM via Glycoprotein A repetitions predominant (GARP) which binds latent TGF‐β1 or Leucine Rich Repeat Containing 32 (LRRC33) in turn binding pro‐TGF‐β1 (Lodyga & Hinz, 2020). It would then be activated from its latency associated peptide (LAP) presented on the lEV surface within the small latent complex (SLC). As we found here, the activated TGF‐β1 is then released and able to bind its cognate receptors (TβRI and TβRII) to promote invasion. SB‐431542, the small molecule inhibitor binding the ATP binding site of TβRI blocks phosphorylation of Smad2/3 and as found previously, but with cardiomyocytes, reduces invasion (Waghabi et al., 2007).

In this current study we have not considered the role of *T. cruzi* EVs. It should be noted therefore that others have studied the effect of *T. cruzi*‐EVs on cell permeability and shown a resulting alteration of actin filaments resulting in increased numbers of parasites per cell (D'Avila et al., 2021; Lovo‐Martins et al., 2018; Moreira et al., 2019). As noted above, others still have also shown *T. cruzi*‐EVs to increase uptake in macrophages preincubated with *T. cruzi* Y strain Mtr supernatant and purified Mtr EVs, in TLR2 and TLR4‐transfected CHO/CD14 cells (Cronemberger‐Andrade et al., 2020). Besides epithelial and fibroblast cells that Mtr interact with at the invasion site, the first immune cells would be macrophages and dendritic cells, so it would also be important going forward, to elucidate lEV biogenesis in these cell types.

## 5 CONCLUSION

This study has shown parasite stimulation through mechanosensitive ion channels (MSCs) leading to Ca^2+^‐mediated lEV release from host cells and that this could be inhibited with the selective cationic MSC inhibitor, GsMTx4. We also showed the importance of store‐activated calcium entry in *T. cruzi*‐mediated lEV release, with a 57%–64% decrease in lEV release in the absence of extracellular calcium, or where SOCE was specifically inhibited, and that store‐independent calcium entry played a minor role. Calpeptin, as well as reducing lEV from Mtr‐stimulated host epithelial cells, likely reduced invasion by inhibiting calpain's ability to remodel the actin cytoskeleton in host cells; actin remodelling is needed for both invasion and lEV release. Restoration of lEV levels from calpeptin‐treated host epithelial cells, demonstrating reduced actin depolymerization, upon stimulation with Mtr, was unable to recover invasion levels. lEVs from parasite‐treated monocytes, however, did increase cellular invasion. We showed lEVs to interact (likely PM fusion‐mediated following protein interaction) with *T. cruzi* metacyclic trypomastigote forms, such that TGF‐β1 is activated from its latent complex on the lEVs and released as the mature 25 kDa homodimer, stimulating TGFβR‐mediated signalling in HeLa cells and promoting invasion.

## AUTHOR CONTRIBUTIONS

**Ephraim A. Ansa‐Addo**: Conceptualization (supporting); data curation (equal); formal analysis (equal); funding acquisition (supporting); investigation (equal); methodology (lead); project administration (supporting); resources (supporting); software (supporting); supervision (supporting); validation (supporting); visualization (supporting); writing—original draft (equal); writing—review and editing (equal). **Paras Pathak**: Conceptualization (supporting); data curation (equal); formal analysis (equal); investigation (supporting); methodology (equal); project administration (supporting); validation (equal); visualization (equal); writing—original draft (supporting); writing—review and editing (supporting). **Izadora Volpato Rossi**: Conceptualization (equal); data curation (equal); formal analysis (supporting); funding acquisition (supporting); investigation (equal); methodology (supporting); project administration (supporting); software (supporting); supervision (supporting); validation (supporting); visualization (equal); writing—original draft (supporting); writing—review and editing (equal). **Maria V. McCrossan**: Data curation(equal); Investigation (equal); Methodology (equal); Resources (equal); Visualization (equal); Writing—review & editing (equal). **Mahamed Abdullahi**: Conceptualization (supporting); data curation (supporting); formal analysis (equal); funding acquisition (supporting); investigation (supporting); methodology (supporting); project administration (supporting); resources (supporting); software (supporting); supervision (supporting); validation (supporting); visualization (equal); writing—original draft (supporting); writing—review and editing (equal). **Dan Stratton**: Conceptualization (supporting); data curation (supporting); formal analysis (equal); funding acquisition (supporting); investigation (supporting); methodology (supporting); project administration (supporting); resources (supporting); software (supporting); supervision (supporting); validation (supporting); visualization (equal); writing—original draft (equal); writing—review and editing (equal). **Sigrun Lange**: Conceptualization (supporting); data curation (supporting); formal analysis (equal); funding acquisition (supporting); investigation (supporting); methodology (supporting); project administration (supporting); resources (supporting); software (supporting); supervision (supporting); validation (supporting); visualization (equal); writing—original draft (equal); writing—review and editing (equal). **Marcel I. Ramirez**: Conceptualization (supporting); data curation (supporting); formal analysis (equal); funding acquisition (supporting); investigation (supporting); methodology (supporting); project administration (supporting); resources (supporting); software (supporting); supervision (supporting); validation (supporting); visualization (equal); writing—original draft (supporting); writing—review and editing (equal). **Jameel M. Inal**: Conceptualization (lead); Data curation(supporting); Formal analysis (equal); Funding acquisition (lead); Investigation (lead); Methodology(supporting); Project administration (equal); Resources (lead); Software (equal); Supervision (lead); Validation (lead); Visualization (equal); Writing—original draft (lead); Writing—review & editing (lead).

## CONFLICT OF INTEREST STATEMENT

The authors report no conflict of interest.

## Supporting information



Supporting Information
