# Supplementary material for: Monocyte‐derived extracellular vesicles, stimulated by Trypanosoma cruzi, enhance cellular invasion in vitro via activated TGF‐β1
Source: J Extracell Vesicles. 2024 Nov 29;13(11):e70014. doi: 10.1002/jev2.70014 (PMC11605483; doi:10.1002/jev2.70014)
Supplement: Supplementary file 1 — Supporting Information [file JEV2-13-e70014-s001.docx]

**Supplementary Figure Legends**

**Fig. S1** Differential expression of Gp90 on metacyclic trypomastigote forms (Mtr) and transmission electron microscopy of *T. cruzi* Mtr releasing extracellular vesicles (EVs). (*A*), Western blotting showing expression of Gp90 in T. cruzi Mtr but not epimastigotes. HeLa cells in 12-well plates were washed in EV-free RPMI, and after 1h preincubation in EV and serum-free RPMI (EVSF-RPMI), cells were placed in EVSF-RPMI/CaCl_2_ (1 mM) for 20 min. (*B* and *C*), metacyclic trypomastigotes releasing plasma membrane-derived EVs (microvesicles (MVs)) indicated by arrowheads and inset . (*D* and *E*), showing an MVB near the flagellar pocket, filled with intraluminal vesicles (which will be released as exosomes upon fusion with the PM). F, flagellum; FP, flagellar pocket, bb, basal body; K, kinetoplast; MVB, multivesicular body; ILVs, intraluminal vesicles; R. reservosome.

**Fig. S2** Characterisation of lEVs released from host cells following interaction with intracellular pathogens. (*A*), Detached HeLa cells (1x10^6^/well in triplicate) were stimulated with *Salmonella enterica* subsp. *enterica* serovar Typhimurium (wild-type, strain SL1-344) (ST) or ST (ΔSP11), a non-invasive mutant strain, at a bacteria-to-cell ratio of 5:1, without or in the presence of EGTA (light green bars), at 5 mM. lEVs released from host cells left non-induced (NI), or treated with sublytic C5b-9 (MAC) in the presence or absence of EGTA were used as controls. (*B*), Similar assays were performed with *Giardia intestinalis*, trophozoite forms, 1×10^6^ HeLa cells/well stimulated with a parasite-to-cell ratio of 5:1. Data represents the mean ± SD of two independent experiments performed in triplicate. ***P*= 0.0043 was considered statistically significant. (*C*), The 15K lEVs isolated from HeLa cells, induced by *T. cruzi* showed, by NTA analysis, a modal peak size of 233 ±29 nm (TEM inset, scale bar, 100 nm). (*D*), The lEVs were positive by western blotting for Tumour Susceptibility Gene 101 protein (TSG101) and annexin A1 (ANXA1). They were also positive for Annexin V (AnV)-FITC staining (89.9 %), (*E*) and 89.0 % positive for CD44 by flow cytometry, (*F*). (*G*), Increased expression of Lamp2 in HeLa cells infected with 10:1 or 5:1, parasite:host ratio (median fluorescence intensity, MFI 72 ±4 versus 36 ±2, respectively) and by western blotting of released EVs, (*H*); TLR4 was also detected on released lEVs by western analysis, (*H*). (*I*), The sEVs isolated from HeLa cells, stimulated by *T. cruzi* showed, by NTA analysis a modal peak size of 95 nm ±17.5 nm (TEM inset, scale bar, 100 nm). These 100K-15K sEVs were positive for TSG101 and ANXA1, (*J*). (*K*), a mixture of Extracellular Vesicles (exosomes and sEVs/lEVs) were applied to a 10-40% sucrose gradient and spun at 30,000 × *g* for 1 h. All fractions were labelled with anti-TSG101-FITC at 10 µg/mL and analysed by flow cytometry. The density of each fraction was determined using a refractometer. Transmission Electron Microscopy examinations of fraction #8 (inset, scale bar 100 nm) shows likely exosomes exhibiting the typical “cup-shaped” morphology. Data represent mean ± SD of two independent experiments performed in triplicate. (*L*), TEM image of MVB fusing with plasma membrane and releasing its cargo or exosomes. (*M*), Total EVs enumerated by NTA from Mtr-infected and uninfected HeLa cells and classified as sEVs (≤125 nm), (*N*) or as lEVs (125-500 nm), (*O*).

**Fig. S3** lEV release and cellular invasion with Mtr-treated HeLa cells is inhibited by RGD peptide and MβCD. HeLa cells (1x10^5^/well for invasion studies and 1x10^6^/well in triplicate for mEV stimulation) infected with Mtr had reduced infection levels with RGD peptide (200 µg/mL) (*A* and *B*) and lEV release, (*C*). MβCD, but not nystatin or filipin, also reduced infection, (*E)* and lEV release (*F*). EGTA (5 mM) was used as a negative control for parasite-stimulated Ca^2+^-mediated lEV release. The inhibitors used to limit *T. cruzi*-mediated lEV release did not work (except with MβCD) when the stimulus was sublytic complement (*D* and *G*). Data represents the mean ± SD of three independent experiments performed in triplicate. **P*< 0.05, ***P*< 0.01 and ****P*< 0.001 were considered statistically significant. For the RGD experiment 4 biological replicates (n=4) and 3 technical repeats (N=3) were performed and data are presented as mean ±SEM.

**Fig. S4** siRNA knockdown of expression of calpain small subunit 1. HeLa cells (1x10^5^/well in triplicate) seeded overnight were washed twice with SF-RPMI and incubated without HiPerfect transfection reagent (HPP), or transfected with control (Ctrl) siRNA or various *CAPNS1* (calpain small subunit 1) siRNA sequences (50 nM) targeting different regions at 37°C for 48 h. (*A*) Immunoblotting analysis of cell extracts (30 µg) after incubation of HeLa cells without HiPerfect transfection reagent (HPP) or transfected with control (Ctrl) siRNA or *CAPNS1* siRNA. Membrane was probed with mouse anti-CAPNS1 antibody (1:500) and mouse anti-actin antibody (1:500) as loading control. (*B-D*) For flow cytometry analysis and immunofluorescence (IF), treated cells were washed twice with D-PBS, trypsinised to suspension or not for IF, and incubated with mouse anti-CAPNS1 antibody (5 µg/mL) in 1% BSA/PBS at 4 °C for 1 h with shaking. Cells were washed three times with cold PBS and incubated with 10 µg/mL of mouse anti-IgG-FITC or mouse anti-IgG-Alexa Fluor® 488 (IF) at 4 °C for 1 h with shaking. After washing three times with cold PBS, samples were analysed by flow cytometry (*B,C*) or fluorescence microscopy (*D*). Mouse anti-IgG-FITC was used as labelling control. Data presented is the mean ± SD of three independent experiments performed in triplicate. **P*= 0.0231, ****P*< 0.0001 were considered statistically significant. Scale bar= 20 µm.

**Fig. S5** Cytokines found in lEVs released from THP-1 monocyte cells infected with *T. cruzi* metacyclic trypomastigote forms. (*A*), cytokine antibody array and densitometry values (net intensities), (*B*) for cytokines found in lysed mEVs (*, p≤0.05; **, p≤0.01) and (*C*), fold changes (>0, values greater in lEVs from infected cells). THP-1 cells (3 x 10^7^) were washed twice, and preincubated in RPMI (1h). Cells were resuspended in fresh RPMI supplemented with 0.5 mM CaCl_2_ and stimulated with *T. cruzi* at a 5:1 ratio or left unstimulated for 30 min at 37 °C and then placed on ice. lEVs were recovered as described in Materials and Methods and washed. lEVs (5 × 10^6^/mL) were lysed with 0.1 %(v/v) Triton X-100 together with protease inhibitors and cytokines measured using a human cytokine antibody array kit (R&D Systems) following the manufacturer’s instructions or by ELISA (*D* and *E*) in which measurements of IL-13 and IFN-γ were performed on lysed lEVs from infected THP-1 cells.
